# Atomically Dispersed Cu Nanozyme with Intensive Ascorbate Peroxidase Mimic Activity Capable of Alleviating ROS‐Mediated Oxidation Damage

**DOI:** 10.1002/advs.202103977

**Published:** 2021-12-23

**Authors:** Yuan Chen, Hang Zou, Bo Yan, Xiaoju Wu, Weiwei Cao, Yihang Qian, Lei Zheng, Guowei Yang

**Affiliations:** ^1^ State Key Laboratory of Optoelectronic Materials and Technologies Nanotechnology Research Center School of Materials Science and Engineering School of Physics Sun Yat‐sen University Guangzhou Guangdong 510275 P. R. China; ^2^ Department of Laboratory Medicine Nanfang Hospital, Southern Medical University/The First School of Clinical Medicine Southern Medical University Guangzhou Guangdong 510515 P. R. China

**Keywords:** antioxidant, ascorbate peroxidase, single‐atom nanozyme, specific activity

## Abstract

Ascorbate peroxidase (APX) as a crucial antioxidant enzyme has drawn attentions for its utilization in preventing cells from oxidative stress responses by efficiently scavenging H_2_O_2_ in plants. For eliminating the specific inactivation of natural APXs and regulating the catalytic activity, single‐atom nanozymes are considered as promising classes of alternatives with similar active sites and maximal atomic utilization efficiency to natural APXs. Herein, graphitic carbon nitride (g‐C_3_N_4_) anchored with isolated single copper atoms (Cu SAs/CN) is designed as an efficient nanozyme with intrinsic APX mimetic behavior. The engineered Cu SAs/CN exhibits comparable specific activity and kinetics to the natural APXs. Based on the density functional theory (DFT), Cu‐N_4_ moieties in the active center of Cu SAs/CN are determined to exert such favorable APX catalytic performance, in which the electron transfer between Cu and coordinated N atoms facilitates the activation and cleavage of the adsorbed H_2_O_2_ molecules and results in fast kinetics. The constructed Cu SAs/CN nanozyme with superior APX‐like performance and high biocompatibility can be applied for effectively protecting the H_2_O_2_‐treated cells against oxidative injury in vitro. These findings report the single‐atom nanozymes as a successful paradigm for guiding nanozymes to implement APX mimetic performance for reactive oxygen species‐related biotherapeutic.

## Graphical abstract

Graphitic carbon nitride anchored with isolated single copper atoms (Cu SAs/CN) is firstly designed as an efficient nanozyme with intrinsic ascorbate peroxidase (APX) mimetic behavior. The engineered Cu SAs/CN exhibits comparable specific activity and kinetics to the natural APXs. Based on the superior APX‐like performance and high biocompatibility, Cu SAs/CN is applied for effectively protecting the H_2_O_2_‐treated cells against oxidative injury in vitro.

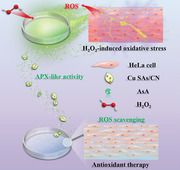

## 1 Introduction

Normally, in the biological system, excess reactive oxygen species (ROS) accumulation often results in pathological damage.^[^ ^1^, ^2^ ^]^ Among the antioxidant enzymes (superoxide dismutase, glutathione peroxidase, and catalase which mainly occur in peroxisomes), ascorbate peroxidase (APX) with a high affinity against H_2_O_2_ plays a crucial role in promoting plant cellular tolerance to oxidative stress through catalyzing the conversion of H_2_O_2_ into H_2_O using ascorbic acid as electron donor.^[^ ^3^, ^4^ ^]^ Though APX functions as the vital regulator for maintaining the ROS balance in plant cells, APX is susceptible to multiple redox‐related post‐transitional modifications (PTMs) and easily inactivated by specific inhibitors through blocking the oxidation of H_2_O_2_ with heme.^[^ ^4^, ^5^ ^]^ Recently much effort has been put into regulating the activities of APXs in terms of post‐transitional modifications of the proteins,^[^ ^6^, ^7^ ^]^ it only succeeded marginally. Until now, for characterizing the regulation by PTMs, only a limited number of proteins have been investigated.^[^ ^8^ ^]^ Meanwhile, PTM involves multiple factors such as the protein structure, the microenvironment where the protein locates, the underlying nitration mechanism.^[^ ^9^ ^]^ The multiple covalent changes lead to complicated effects (gain or loss in protein function or invariant in function). Of note, PTM is still facile challenged by the inactivation of APXs at the molecule level. Therefore, it is of great importance to explore robust artificial alternatives with similar or even superior performance.

Nanozyme defines a generation of nanomaterials with enzyme‐like behavior.^[^ ^10^, ^11^, ^12^ ^]^ It combines the advantages of nanomaterials (easy preparation, low cost for scale‐up, and especially high stability) that catalyze biochemical reactions under ambient conditions.^[^ ^13^, ^14^, ^15^, ^16^ ^]^ In particular, the single‐atom nanozymes with atomically dispersed active metal centers are recently considered as a novel frontier due to their maximum utilization efficiency of metal atoms and high activity.^[^ ^17^, ^18^, ^19^, ^20^, ^21^, ^22^ ^]^ To our best acknowledge, ascorbate oxidase (AO) and APX are Cu and Fe containing metalloenzymes involves in redox reactions. The progression of the reactions is determined by the reactivity of the metals.^[^ ^23^ ^]^ Because single‐atom nanozymes possess similar metal—N*_x_* active sites to natural metalloenzymes,^[^ ^11^, ^24^ ^]^ it has been hypostasized as promising classes of alternatives to mimic the center structure and catalytic behavior of APXs. Up to now, the prussian blue nanoparticles^[^ ^25^ ^]^ and the Ru‐based nanoparticles have been reported to mimic the functionalities of APX owing to the high activity of the center metals.^[^ ^26^ ^]^ Nevertheless, the instability and cytotoxicity of ferricyanide under physiological conditions, high cost, and low atom utilization efficiency of noble metal atoms further limit their utilizations in the aspect of cellular and biotherapeutic. In addition, the discussion on the specific reaction mechanism of nanozymes as APX mimics is meager and yet to be enlightened.

In terms of these issues, Cu‐based single‐atom nanomaterials with the low‐cost earth‐abundant element, outstanding biocompatibility, and similar active centers to the natural APXs have been targeted as the initial paradigm for gaining insight into the APX mimetic reaction process and the origin of the APX activity from single‐atom nanozymes. In this article, we constructed the atomically dispersed nanozyme Cu SAs/CN with Cu—N_4_ moieties active sites and initially demonstrated its comparable APX mimetic activity and fast kinetic compared with the natural APXs. Based on the experimental studies and theoretical calculations, the specific APX‐like reaction process and the correlation between the well‐defined active sites and such favorable catalytic activity have also been explored. They identified that the superior APX‐like activity of Cu SAs/CN families from the electron transfer process between the single Cu atoms and coordinated N atoms, which further activates the absorbed H_2_O_2_ and leads to the facile cleavage. The Cu SAs/CN nanozyme exhibits robust biocompatibility and effective antioxidant capability under oxidation stress. Therefore, these findings build a novel platform to enable the utilization of single‐atom nanozyme as a promising APX alternative for reactive oxygen species (ROS)‐related biotherapeutic.

## 2 Results and Discussions

### 2.1 Preparation and Characterization of Cu SAs/CN

**Scheme 1:**
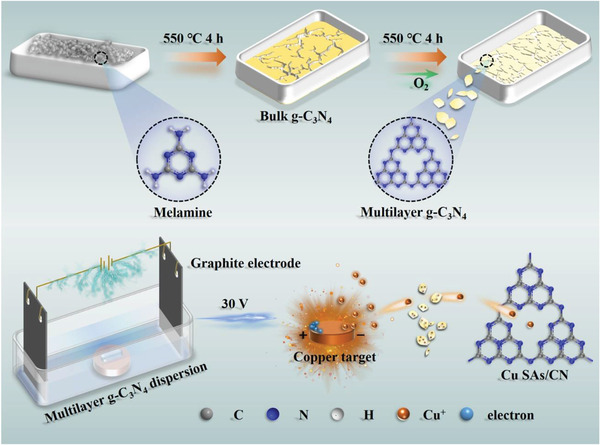
Schematic illustration of the synthesis of Cu SAs/CN.

**Figure 1:**
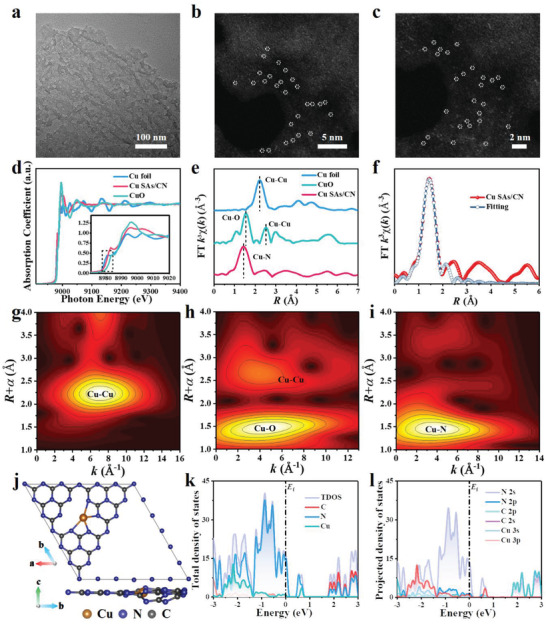
Morphology, atomic structure characterization, and electronic structure analysis of Cu SAs/CN. a) TEM image. b,c) AC HAADF‐STEM images of Cu SAs/CN. The Cu single atoms are highlighted in white cycles. d) Normalized X‐ray absorption near‐edge structure (XANES) spectra of Cu foil, CuO, and Cu SAs/CN. Inset is the enlarged image of the dashed box. e) Fourier transform extended X‐ray absorption fine structure (EXAFS) spectra at the Cu K‐edge. f) The corresponding EXAFS fitting curves of Cu SAs/CN in R space. g–i) Wavelet transform (WT) spectra of Cu K‐edge for Cu foil, CuO, and Cu SAs/CN, respectively. j) The optimized unit structure of Cu SAs/CN (top view and side view). The blue, gray, and orange balls refer to the N, C, and Cu atoms, respectively. k) Total density states and orbital distribution of each element in Cu SAs/CN. l) Projected density states of Cu 3s, Cu 3p, C 2s, C 2p, N 2s, and N 2p orbital distribution.

The Cu SAs/CN nanozymes were fabricated via an ingenious and simple electrochemical deposition method (as illustrated in **Scheme** **1**), in which Cu atoms from the copper target were stripped into single atoms and anchored with the graphitic carbon nitride (g‐C_3_N_4_). As shown by the scanning electron microscopy (SEM), transmission electron microscopy (TEM), and high angle annular dark‐field scanning TEM (HAADF‐STEM) images in Figures S1 and S2 (Supporting Information) and **Figure** **1**a, Cu SAs/CN was prepared with a similar irregular ultrathin‐layer porous morphology to CN. Energy dispersive spectroscopy (EDS) analysis in Figure S1 (Supporting Information) suggests that Cu, C, and N elements are homogeneously distributed on the framework of Cu SAs/CN. According to the inductively coupled plasma‐atomic emission spectrometry (ICP‐AES) analysis, the loaded Cu contents of Cu SAs/CN‐30, Cu SAs/CN‐60, and Cu SAs/CN‐90 (the electrochemical deposition process last for 30, 60, and 90 min) are determined to be 0.18, 0.31, and 0.48 wt%, respectively. The well‐defined crystalline structure of Cu SAs/CN samples from the X‐ray diffraction (XRD) patterns in Figure S3 (Supporting Information) is in great agreement with CN, indicating that the anchoring of Cu SAs barely changes the host structure. To further obtain the direct information of the atomic structure of Cu SAs/CN, the aberration‐corrected HAADF‐STEM (AC HAADF‐STEM) analysis is also conducted (Figure 1b,c and Figure S4, Supporting Information). The individual bright spots were founded in AC HAADF‐STEM images (uniformly dark dots for the bright‐field). Besides, the majority of the bright spots are below 0.2 nm in diameter (Figure S4, Supporting Information), demonstrating that Cu elements were uniformly distributed over the CN matrix as single atoms.^[^ ^12^, ^19^, ^27^ ^]^ Meanwhile, no discernable bulk‐like crystalline copper species are founded in XRD patterns or Fourier transform infrared (FTIR) spectroscopy (Figure S5, Supporting Information), further proving the atomic distribution of Cu atoms. The Brunauer–Emmett–Teller (BET) measurements in Figure S6 (Supporting Information) further manifest that Cu SAs/CN possessed a porous structure with a large surface area compared with CN, which is conducive to exposure the Cu active sites.

### 2.2 Surface Structure Analysis of Cu SAs/CN by X‐Ray Photoelectron and X‐Ray Absorption Fine Structure Spectroscopy

The information on surface chemical compositions and atomic structure of CN and Cu SAs/CN was studied by X‐ray photoelectron spectroscopy (XPS) and X‐ray absorption spectroscopy (XAS). Only C, N, O, and Cu elements are observed from the survey XPS spectra in Figure S7 and Table S1 (Supporting Information) which conforms with the EDS results. As depicted in the high‐solution N 1s spectra, the XPS signal of CN can be deconvoluted into three peaks, assigning to the pyridinic N═C—N (398.2 eV), N(C)_3_ (399.6 eV), and C—N—H (400.8 eV),^[^ ^28^ ^]^ respectively. Compared with CN, the binding energy and intensity of pyridinic N 1s (Cu SAs/CN) are decreasing marginally. Moreover, a new peak (399.0 eV) corresponds to the Cu—N bond are observed in Cu SAs/CN samples, suggesting that Cu atoms are mainly coordinated with pyridinic N atoms to form the Cu—N moieties.^[^ ^29^ ^]^ Besides, the two distinct peaks at 932.3 and 952.3 eV in the high‐solution Cu 2p XPS spectra are ascribed to the Cu 2p_3/2_ and Cu 2p_1/2_ of Cu^+^, implying Cu^+^ species are the predominant oxidation state in Cu SAs/CN.^[^ ^30^, ^31^ ^]^

The X‐ray absorption near‐edge structure (XANES) and the extended X‐ray absorption fine structure (EXAFS) were also conducted to explore the coordination environment of Cu atoms. In Figure 1d, the XANES of Cu SAs/CN shows a similar edge shape with Cu foil and CuO reference. And the absorption edge position of Cu SAs/CN is situated between those of Cu foil and CuO, indicating the existence of Cu^+^ in Cu SAs/CN (Cu*^*δ*^* ^+^, 0 < *δ* < 2). As shown in Figure 1e, the Fourier transform (FT) of the Cu K‐edge EXAFS spectra of Cu SAs/CN is quite different from those of the bulk references of Cu foil and CuO. A dominant EXAFS FT peak founded in the related fitting curves of Cu SAs/CN around 1.44 Å can be ascribed as the Cu—N scattering path.^[^ ^30^, ^32^, ^33^ ^]^ Whereas no Cu—Cu coordination peak at 2.2 Å can be observed, suggesting that Cu exists as an isolated atom that is atomically dispersed on the CN matrix.^[^ ^29^ ^]^ Meanwhile, the EXAFS fitting curves perfectly reproduced the experimental EXAFS FT experimental curves of Cu SAs/CN (Figure 1f and Figure S8, Supporting Information), Cu foil and CuO (Figure S9, Supporting Information). The structure parameters and quantitative chemical configuration of Cu atoms were then achieved by fitting the EXAFS at the Cu K‐edge (Table S2, Supporting Information). The average coordination number of Cu SAs/CN is then determined to be nearly 4 based on the quantitative EXAFS fitting analysis in *k* and *R* space. Because the similar bond length of the Cu—N and Cu—O,^[^ ^34^ ^]^ for further discriminating the N and O signals of Cu—N in *k* space, wavelet transform (WT) analysis had also been carried out to support the FT results. From the WT contour patterns in Figure 1g–i, Cu SAs/CN shows one intensity maximum at 4.0 Å attributed to the Cu—N coordination. In comparison with the WT plots of Cu foil and CuO, no intensity maximum assigned to the Cu—Cu can be founded. Together, the above results accentuate that Cu SAs/CN nanozymes with atomically dispersed Cu—N_4_ moieties are successfully prepared.

To exploring the electronic structure of Cu SAs/CN, the density functional theory (DFT) calculations were also conducted. Combing with the EXAFS results, the single Cu atom is anchored on CN and coordinated with N atoms (Figure 1j). According to the total density of states (TDOS) profiles of Cu SAs/CN in Figure 1k, the top valence band (VB) primarily stems from the orbitals of N, suggesting that the single Cu atom is covered by the localized electrons from N atoms.^[^ ^35^ ^]^ To gain deeper insight into the orbital hybridization between Cu and N atoms, the projected density of states (PDOS) profiles have also been calculated (Figure 1l). Obvious strong overlaps between N 2p and Cu 3d orbitals can be observed with that of pure CN (Figure S10, Supporting Information). Such a tight electronic interaction (Cu—N) enables the anchoring of single Cu atoms on CN. Additionally, the corresponded differential charge distribution maps in Figure S11 (Supporting Information) further prove the electron transfer between Cu and the coordinated N atoms, in which a remarkable increase of electron density is founded at the neighboring N atoms with a decreased electron density of Cu atom. Meanwhile, the Hirshfeld charge analysis in Figure S11 (Supporting Information) presents that the anchoring of atomically dispersed Cu atoms will lead to an electron transition through the Cu atom to the linked N atoms when compared with the pure CN. Such a charge transfer channel from Cu—N_4_ coordination moieties can improve the charge mobility of CN.^[^ ^30^ ^]^ The fast charge transfer may allow more available charges for driving the catalysis reaction.^[^ ^36^ ^]^ Similar trends can also be observed from the electrochemical measurements in Figure S12 (Supporting Information).

### 2.3 Investigation for the APX‐Like Behavior of Cu SAs/CN

**Figure 2:**
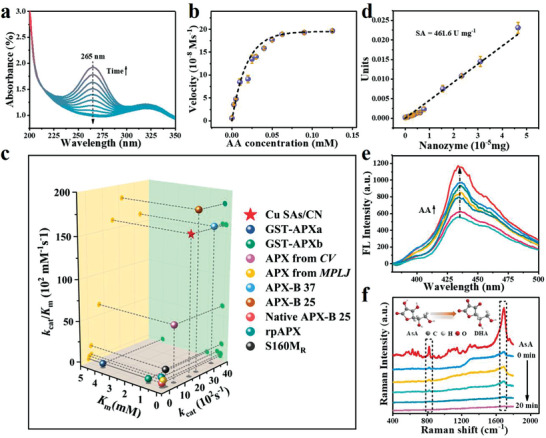
Characterizations of the APX‐like performance of Cu SAs/CN. a) Time‐dependent ultraviolet–vis (UV–vis) absorption spectra in the presence of Cu SAs/CN, AsA, and H_2_O_2_. b) Michaelis–Menton curves by varying AsA concentration at a constant concentration of H_2_O_2_. c) The specific activities (SA) of Cu SAs/CN. The SA is determined by plotting the APX‐like activities against the weight. SA value is obtained from the slope of the resultant straight lines. d) Comparison of the kinetic constants (*K*_m_, *k*_cat_, and *k*_cat_/*K*_m_) based on Cu SAs/CN, natural APXs, and APX mutants for AsA substrate. e) Fluorescence spectra of OPDA‐Cu SAs/CN with increasing concentrations of AsA. f) The in situ Raman spectra during APX‐like catalytic oxidation of AsA molecules on Cu SAs/CN in the presence of H_2_O_2_ at different time intervals. Inset image reflects the oxidation of AsA molecules by Cu SAs/CN with the presence of H_2_O_2_.

Generally, the APX‐like activity is assayed by monitoring the consumption of ascorbic acid (AsA). As presented in Figure S13 (Supporting Information) and **Figure** **2**a, the characteristic absorption peak of AsA is around ≈265 nm. When incubated with either H_2_O_2_ and CN, the absorbance of AsA shows imperceptibly fluctuation, whereas, decreases dramatically over time upon being treated with Cu SAs/CN. AsA is rapidly depleted less than 5 min. These results suggesting that CN has no functionality on depleting AA in the presence of H_2_O_2_ through APX mimetic way. In particular, to further discriminate the active sites of the APX‐like performance of Cu SAs/CN, the thiocyanate ions (SCN^–1^) poisoning experiments have been taken into account where SCN^–1^ is widely known to poison the metal‐centered catalytic sites.^[^ ^37^, ^38^ ^]^ It was founded that the APX‐like activity of Cu SAs/CN was seriously inhibited in introducing SCN^–1^ into the system (Figure S14, Supporting Information). Besides, the relative activity distinctly with the increasing concentrations of SCN^–1^, convincing that the assumption of Cu—N_4_ moieties in Cu SAs/CN rather than the CN matrix as the active centers for mimicking the APX activity.^[^ ^24^ ^]^ To avoid the interference of some nucleic acids and amino acids with a high absorption value around 260–280 nm, we applied 290 nm (with an absorbance coefficient of 2800 mm ^–1^ cm^–1^) in place of 265 nm as ascorbate was oxidized for further steady‐state kinetic analyzing.^[^ ^3^, ^26^ ^]^ Similar to the natural APX, the catalytic activity of Cu SAs/CN also shows pH‐ and temperature‐dependent (Figure S15, Supporting Information). pH 7.4 and 25 °C were further adopted as the standard conditions for subsequent analysis of Cu SAs/CN activity. As depicted in Figure 2b and Figures S16 and S17 (Supporting Information) with the addition of H_2_O_2_, the decomposition of AsA catalyzed by Cu SAs/CN followed the Michaelis–Menten kinetics and ping‐pong mechanism^[^ ^39^, ^40^ ^]^ (Figure S18, Supporting Information). The apparent Michaelis–Menten constant (*K*_m_) of Cu SAs/CN is about 100–400 times lower than that of the natural APX^[^ ^41^, ^42^, ^43^, ^44^, ^45^ ^]^ (Figure 2c and Tables S3 and S4, Supporting Information), implying an extremely strong affinity between Cu SAs/CN and AsA. From the estimation of *V*_max_, the *k*_cat_ and *k*_cat_/*K*_m_ values (turnover number) of Cu SAs/CN were then determined as 1.60 × 10^2^ and 1.60 × 10^4^ for AsA, which was one or three orders of magnitude higher than those of the APX from *Musa paradisaca* leaf, the Wild APX and APX mutants.^[^ ^43^, ^46^ ^]^ Especially, Cu SAs/CN performs a comparable specific activity (SA, about 461.6 U mg^–1^) with natural purified APXs (Figure 2d and Table S5, Supporting Information). Thus, these results confirm that Cu SAs/CN not only improve the affinity for AsA, but also exerted a critical influence on the catalytic activity and efficiency as APX mimics. Besides, the recycling (Figure S19, Supporting Information) and selective tests (Figure S20, Supporting Information) also suggested that the sufficient robustness and selectivity of the APX‐like activity of Cu SAs/CN, outperforming natural APXs which are facile susceptible by the harsh environment.

Generally, natural APXs participate in the ascorbate‐glutathione cycle, reduce the H_2_O_2_ to H_2_O with AsA as substrate and convert AsA to the oxidized form of monodehydroascorbate (MDHA). MDHA is further oxidized into hydroascorbate (DHA)^[^ ^5^ ^]^ (Figure S21, Supporting Information). As noted above, to explore the possible mechanism of intrinsic APX mimetic performance of Cu SAs/CN, fluorescence and in situ Raman probes were conducted to detect the final product (DHA) of the AsA‐Cu SAs/CN‐H_2_O_2_ system. The nonfluorescent OPDA is capable to track DHA and form 3‐(1,2‐dihydroxyethyl) furo[3,4b]‐quinoxaline (DFQ) with a strong emitting fluorescence at 425 nm.^[^ ^47^, ^48^ ^]^ As shown in Figure 2e, a fluorescence peak with an emission maximum at 430 nm is observed in the OPDA‐Cu SAs/CN‐AsA‐H_2_O_2_ system. Furthermore, the fluorescence intensity is enhanced and linear with AsA concentration (Figure S22, Supporting Information). The above results verify that Cu SAs/CN catalyzed the oxidation of AsA with H_2_O_2_ to generate DHA. Besides, the Raman scattering technique is also performed for in situ monitoring the oxidation of AsA molecule which is confined on the surface of Cu SAs/CN at different intervals. The oxidation process was obtained by comparing the Raman spectra between the AsA solution and reaction solution in real‐time (Figure 2f). The three distinct Raman peaks around 1692.6 and 823.3 cm^–1^ are indexed to the C═C and C—C—O vibration bands of AsA (Table S6, Supporting Information), respectively. Whereas, the C═C and C—C—O vibration peaks of the reaction solution nearly disappeared in about 20 min, confirming that the surface target AsA molecules are rapidly converted to their oxidized form DHA by Cu SAs/CN. Taken together, combined with the ping‐pong kinetics (Figure S18, Supporting Information), the possible mechanism for Cu SAs/CN as APX mimics can be expressed as

(1) $$2\mathrm{AsA}+H_{2}O_{2}\overset{\mathrm{Cu}\;\mathrm{SAs}/\mathrm{CN}}{\to }2\mathrm{MDHA}+2H_{2}O$$

Cu SAs/CN first binds with H_2_O_2_, which in turn oxidizes two AsA molecules through two successively electron transfer processes to form MDHA.

### 2.4 DFT Studies on the APX Mimetic Activity of Cu SAs/CN

To further explore the origin of such promising catalytic activity and efficiency of Cu SAs/CN as APX mimics, the DFT calculations were carried out for the H_2_O_2_ molecules reduction process on CN and single‐atom metal centers with AsA as the electron donor. The optimized Cu SAs/CN model is constructed based on the EXAFS data (Figure 1j).

Typically, the absorption energies between H_2_O_2_ molecules and metals can be taken as convenient descriptors for further investigating the dominating enzymatic activities.^[^ ^19^, ^49^, ^50^ ^]^ Compared with pure CN (*E*_ads_: −0.64 eV), Cu SAs/CN with more negative absorption energy (*E*_ads_: −0.94 eV) against H_2_O_2_ is more energetically favorable for the selective absorption of H_2_O_2_. The strong absorption of H_2_O_2_ by Cu SAs/CN weakens the O—O bond and further give rise to the bond distance of O—O (Cu SAs/CN: 1.48 Å, free H_2_O_2_ molecule: 1.2 Å). The weakening of the O—O bond might be contributed to the electrons transfer through the electron push effect of coordinated N atoms in Cu SAs/CN structure during the absorption process.^[^ ^17^, ^49^ ^]^

**Figure 3:**
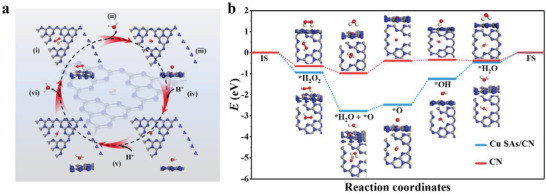
DFT investigation of APX‐like activity over Cu SAs/CN and CN. a) The possible reaction pathway for the reduction of H_2_O_2_ with optimized adsorption configurations on Cu SAs/CN. The blue, gray, white, and orange balls refer to the N, C, H, and Cu atoms, respectively. b) Energy profile diagram for H_2_O_2_ reduction on CN and Cu SAs/CN with AsA as APX substrate.

Meanwhile, it is considered that AsA can be oxidized to MDHA and H_2_O by H_2_O_2_ on the active center of the Cu atom from the above APX mimetic assay (Equation (1)). **Figure** **3**a,b have plotted the energy profile diagram of the most favorable pathway for the reduction of H_2_O_2_ on APX mimic with AsA as substrate. CN and Cu SAs/CN go through a two‐electron reaction process: H_2_O_2_ → * H_2_O_2_ (i) → * H_2_O +*O (ii) → *O (iii) → *OH (iv) → *OH +*H (v) → *H_2_O (vi)→ H_2_O. Asterisk (*) refers to the adsorption sites on the metal surface. The Cu—N_4_ moieties from Cu SAs/CN first bind with H_2_O_2_, and spontaneously dissociate H_2_O_2_ into the intermediate (*H_2_O + *O) via a heterolytic route^[^ ^18^, ^51^ ^]^ (Figure S23, Supporting Information). Comparatively, the easier adsorption and cleavage of H_2_O_2_ on Cu SAs/CN (*E*_a_: −1.83 eV) than CN (*E*_a_: −0.34 eV) agree well with the former APX‐like profile results. After releasing one H_2_O and generating the Cu═O intermediate, an H atom from one AsA molecule is facilely abstracted to form Cu—OH and MDHA on Cu—N_4_ active centers (iv). Notably, the desorption of the H_2_O_2_ with small uphill energy of 0.30 eV for Cu SAs/CN is determined to be the rate‐determining step in the APX mimetic catalysis. Following abstract another H atom from the second AsA molecule, the structure of Cu‐OH forms another MDHA molecule and H_2_O (v and vi). Taken together, the possible reaction process based on DFT are as follows

(2) $$\mathrm{CN}-\mathrm{Cu}\left(I\right)+H_{2}O_{2}\to \mathrm{CN}-\mathrm{Cu}\;\left(\mathrm{II}\right)-O^{+}+H_{2}O$$

(3) $$\mathrm{CN}-\mathrm{Cu}\left(\mathrm{II}\right)-O^{+}+\mathrm{AsA}\to \mathrm{CN}-\mathrm{Cu}\left(\mathrm{II}\right)-\mathrm{OH}+\mathrm{MDHA}$$

(4) $$\mathrm{CN}-\mathrm{Cu}\left(\mathrm{II}\right)-\mathrm{OH}+\mathrm{AsA}\to \mathrm{CN}-\mathrm{Cu}\left(I\right)+\mathrm{MDHA}+H_{2}O$$

To sum up, the structure in which the active single Cu atoms coordinated with N atoms from CN not only activates the H_2_O_2_ molecules and benefits the cleavage of O‐O bond, but also favors the optimization of the energy of each state during the APX‐like process. These further endows Cu SAs/CN with superior activity as APX mimics.

### 2.5 Cytotoxicity and In Vitro Antioxidant Capacity of Cu SAs/CN from H_2_O_2_‐Induced Oxidative Stress

Inspired by the superior APX‐like performance of Cu SAs/CN which can directly scavenge the oxidative H_2_O_2_, the cytotoxicity and in vitro assays were successively conducted based on HeLa cell models to evaluate its antioxidant capacity.

**Figure 4:**
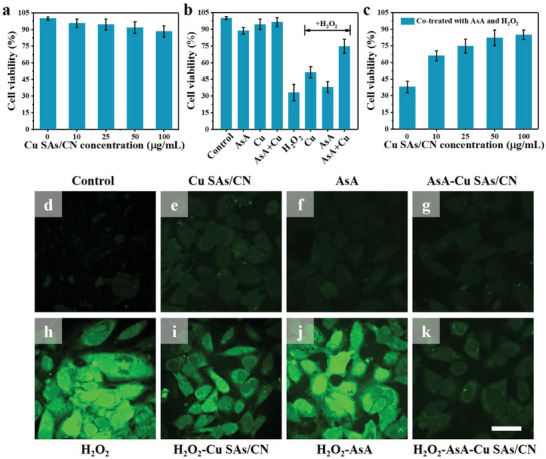
Cytotoxicity and in vitro antioxidant capacity of Cu SAs/CN on HeLa cells. a) Cytotoxicity of Cu SAs/CN at different concentrations on HeLa cells. b) Cell viabilities of HeLa cells pretreated with different systems and c) H_2_O_2_‐AsA at different concentrations of Cu SAs/CN for 24 h. d–k) Effect of Cu SAs/CN nanozyme on ROS generation in HeLa cells induced by H_2_O_2_. The antioxidant capability of Cu SAs/CN was determined by monitoring the DCFH‐DA fluoresce intensities of HeLa cells. Scale bar: 50 µm. (The concentrations of Cu SAs/CN, H_2_O_2_, and AsA are 25 µg mL^–1^, 500 × 10^−6^ m, and 0.0625 × 10^−3^ m, respectively)

The cytotoxicity of Cu SAs/CN was initially evaluated by MMT assay. After 24 h incubation (**Figure** **4**a), the cell viability of HeLa cells maintains more than 90% with increasing concentrations of Cu SAs/CN (0 to 100 µg mL^–1^). Besides, Cu SAs/CN also shows superior stability in Dulbecco's modified Eagle medium (DMEM) in Figure S24 (Supporting Information). These results suggest the remarkable biocompatibility and stability for in vivo applications. For the in vitro antioxidant assay, the effect of pure H_2_O_2_ on HeLa cells was first evaluated. As shown in Figure 4b, the cell viability dramatically drops to 35% after being treated with H_2_O_2_ (500 × 10^−6^ m), which can be attributed to the ROS‐mediated oxidative attack induced by H_2_O_2_.^[^ ^2^ ^]^ Whereas, after coincubation with AsA, the ROS level slightly decreases in these cells, revealing the cytoprotective effect of AsA. Notably, the cell viability recovers from 35% to 51% and 85% in those cells pretreated with Cu SAs/CN or Cu SAs/CN‐AsA. In addition, the recovery of cells exhibits a dosage‐dependent behavior (Figure 4c), further confirming the superior ROS‐scavenging ability and antioxidant capability of Cu SAs/CN as APX mimics. Moreover, the 2,7‐dichlorofluorescein diacetate (DCFH‐DA) assay was also applied for visually assessing the generation of intracellular ROS, in which the DCFH‐DA probe enters into cells and forms 2,7‐dichlorofluorescein (DCF) with green fluorescence after being exposed to ROS.^[^ ^40^ ^]^ As depicted in Figure 4d–k, both of the control or the cells treated with Cu SAs/CN, AsA and Cu SAs/CN‐AsA display weak fluorescence. This phenomenon mainly contributed to the generation of the small amount of ROS stimulated by the introduction of a low dosage of Cu SAs/CN and AsA in HeLa cells.^[^ ^52^, ^53^ ^]^ Nevertheless, intensive green fluorescence can be observed in the cells incubated with pure H_2_O_2_, implying the presence of a high level of ROS. By contrast, the bright fluorescence signal of cells declined after being cultured with nanozyme treatment (Cu SAs/CN‐AsA), indicating that Cu SAs/CN with APX‐like behavior can effectively inhibit the oxidation stress raised by intracellular ROS. Besides, the cell uptake assay in Figure S25 (Supporting Information) further demonstrated that Cu SAs/CN can be internalized by HeLa cells. In addition, Cu SAs/CN is applied to protect the cells by its function inside of cells.

The above results collectively convey the extraordinary ROS elimination capacity of Cu SAs/CN, making them promising antioxidant drug candidates for reducing the H_2_O_2_‐induced oxidative stress.

## 3 Conclusions

In summary, a single‐atom nanozyme with atomically dispersed Cu active sites (Cu SAs/CN) was designed. We for the first time reported that the constructed Cu SAs/CN nanozyme exhibits unique APX‐like activity, which is capable of regulating the intracellular level of H_2_O_2_ as a representative peroxidase. Especially, Cu SAs/CN with comparable catalytic activity (SA = 461. 60 U mg^–1^), substrate affinity (*K*_m_ = 0.01 × 10^−3^ m), and high catalytic efficiency (*k*_cat_/*K*_m_ = 1.60 × 10^4^ mm ^–1^ s^–1^) compared to the natural APX (SA = 918.0 U mg^–1^, *K*_m_ = 0.11 × 10^−3^ m, *k*_cat_/*K*_m_ = 3.10 × 10^2^ mm ^–1^ s^–1^) is considered as a high‐efficient APX alternative. Based on the experimental profiles and theoretical calculations, the APX reaction process and underlying mechanism of the superior catalytic performance have also been investigated. The Cu—N_4_ moieties in the active center of Cu SAs/CN are responsible to exert superior APX‐like catalytic performance, in which the electron transfer between Cu single atoms and coordinated N atoms is beneficial for the activation and cleavage of the adsorped H_2_O_2_ molecules. This also leads to low energy barriers during the APX‐like process, results in efficient kinetics, and favors the optimization of the energy of each state. The in vitro experiments based on HeLa cells further confirm that Cu SAs/CN with intrinsic APX‐like activity possess robust biocompatibility and can impressively protect the H_2_O_2_‐treated cells from oxidation stress. In a word, the present results provide a new perspective to investigate the mechanism of the APX‐like reaction process for nanozymes and build a novel platform to gain insight into the emerging field of single‐atom nanozymes. In addition, it also inspires the specific utilization of nanozymes as APX mimic in ROS‐related biotherapeutic fields.

## Conflict of Interest

The authors declare no conflict of interest.

## Supporting information



Supporting Information

## Data Availability

The data that supports the findings of this study are available in the article and in the supplementary material of this article.
