# Supplementary material for: Atomically Dispersed Cu Nanozyme with Intensive Ascorbate Peroxidase Mimic Activity Capable of Alleviating ROS‐Mediated Oxidation Damage
Source: Adv Sci (Weinh). 2021 Dec 23;9(5):2103977. doi: 10.1002/advs.202103977 (PMC8844488; doi:10.1002/advs.202103977)
Supplement: Supplementary file 1 — Supporting Information [file ADVS-9-2103977-s001.pdf]

ADVANCED SCIENCE
Open Access

Supporting Information

for *Adv. Sci.,* DOI: 10.1002/advs.202103977

Atomically dispersed Cu nanozyme with intensive ascorbate peroxidase mimic activity capable of alleviating ROS-mediated oxidation damage

Yuan Chen[1,†], *Hang Zou*[2,†], *Bo Yan*[1], *Xiaoju Wu*[1], *Weiwei Cao*[1], *Yihang Qian*[1], *Lei Zheng*[2,]* and *Guowei Yang*[1,]*

Supporting Information for

Atomically dispersed Cu nanozyme with intensive ascorbate peroxidase mimic activity capable of alleviating ROS-mediated oxidation damage

Yuan Chen[1,†], Hang Zou[2,†], Bo Yan[1], Xiaoju Wu[1], Weiwei Cao[1], Yihang Qian[1], Lei Zheng[2,*] and Guowei Yang[1,*]

[1]*State Key Laboratory of Optoelectronic Materials and Technologies, Nanotechnology Research Center, School of Materials Science & Engineering, School of Physics, Sun Yat-sen University, Guangzhou 510275, Guangdong, P. R. China.*

[2]*Department of Laboratory Medicine, Nanfang Hospital, Southern Medical University/The First School of Clinical Medicine, Southern Medical University, Guangzhou 510515, Guangdong, P. R. China*

[†] *These authors contributed equally to this work.*

* *Corresponding authors: stsygw@mail.sysu.edu.cn, nfyyzhenglei@smu.edu.cn*

Experimental Section

Chemicals and reagents

Melamine, ascorbic acid (AsA), phosphate buffer saline (PBS, pH = 7.4), *o*-phenylenediamine (OPDA), 3,3’,5,5’-Tetramethylbenzidine (TMB), potassium thiocyanate (KSCN), Vitamin B1 (VB1), riboflavin (VB2), nicotinamide (VB3), Vitamin B6 (VB6) were purchased from Shanghai Aladdin Reagent Co., Ltd. Ethanol, hydrogen peroxide (30%, H_2O_2), acetic acid (HAc), sodium acetate (NaAc), sodium carbonate (Na_2CO_3) and sodium bicarbonate ($NaHCO_3$) were brought from Guangzhou Chemical Reagent Factory. The Copper target (Cu, 99.99%) was achieved from 3A Chemicals Industry. Millipore water (18.2 MΩ) was used throughout the experiments.

Preparation of the matrix g-C_3N_4

In a typical procedure, melamine (10 g) was added to an alumina porcelain boat and then placed in a muffle furnace to raise the temperature to 550 ºC with a heating rate of 10 ºC min^{-1} over 4 h. The obtained agglomerated was then ground into powder (bulk g-C_3N_4). Subsequently, the as-prepared bulk g-C_3N_4 (1 g) was heated to 500 ºC for 4h at 10 ºC min^{-1} under air atmosphere in a muffle furnace to achieve the multilayer g-C_3N_4. Finally, the multilayer g-C_3N_4 was dispersed in high-purity de-ionized water and sonicated. The exfoliated dispersion (0.5 mg mL^{-1}) was labeled as the matrix g-C_3N_4 (CN).

Synthesis of the single Cu atoms/ CN nanozymes (Cu SAs/CN)

The Cu SAs/CN were synthesized by a simple and idiographic electrochemical

deposition technique which has been previously reported by Yan et al.[1-2] As depicted in Scheme 1, in the preparation of Cu SAs/CN, two parallel cleaned graphite flakes with the size of 2*4 cm^2 were employed as the cathode and anode. Both of the electrodes were immersed into a glass chamber with a separation of approximately 10 cm. Under application of a working voltage of 30 V for 30, 60 and 90 min, the samples (labeled as Cu SAs/CN-30, Cu SAs/CN-60 and Cu SAs/CN-90, respectively) were collected from the deposition bath containing the as-prepared CN dispersion (0.5 mg mL^{-1}), a magneton and copper target (99.99%) with a diameter of 25 mm at the center of the bath.

Apparatus and characterizations

The crystal structure was determined by an X-ray diffractometer (XRD, D/Max-IIIA, Rigaku) with Cu Kα radiation (40 kV, 20 mA) with a speed of 4° s^{-1}. The transmission electron microscope (TEM), scanning transmission electron microscope (STEM) and The high-angle annular dark-field scanning transmission electron microscopy (HAADF-STEM) images were acquired using a Spherical aberration-corrected transmission electron microscope (TEM, JEM-ARM200F) with a field-emission gun and an accelerating voltage of 200 kV. Scanning electron microscopy (SEM) images were recorded on a Field-Emission Scanning electron microscopy (SEM, Zeiss Gemini SEM 500) coupled with an energy dispersive X-ray spectrometer (EDX, Bruker X-flesh 5060f). X-ray photoelectron spectra (XPS) were performed on an X-ray photoelectron spectrometer (Thermo-VG Scientific ESCALAB 250). Fourier transform infrared (FTIR) spectra were obtained by an FTIR spectrometer (Frontier,

PerkinElmer Company). Brunauer-Emmett-Teller (BET) specific area was determined by the nitrogen adsorption-desorption isotherms on a Micromeritics (ASAP 2020M) system after vacuum degassing at 120 ºC for 6 h. The gravimetric of copper was evaluated by an inductively coupled plasma-atomic emission spectrometry (ICP-AES, IRIS(HR)). UV-vis absorbance spectra were acquired by a spectrophotometer (UV-3600, SHIMADZU Corporation). The dynamic light scattering (DLS) and the Zeta potential measurements were carried out on an EliteSizer nanoparticle size-Zeta potential and molecule weight analyzer.

X-ray absorption fine structure (XAFS)

The XAFS including the X-ray absorption near-edge structure (XANES) and Fourier transform extended X-ray absorption fine structure (EXAFS) analysis at Cu K-edge were conducted to investigate the coordination environment of Cu atoms on the CN. Cu K-edge XAFS spectra were collected on the BL14W1 beamline at the Shanghai Synchrotron Radiation Facility (SSRF), which using a Si (111) double-crystal monochromator to reduce the harmonic component of the beam. Cu foil and CuO were employed as the reference samples. The obtained XAFS data was then processed in Athena (version 0.9.26) for background-subtracting, normalizing and Fourier transforming. The k^3 weighting, k-range of 3-12 $Å^{-1}$ and R range of 1-3 Å were used for the curve fitting of the Cu foils. The k-range of 2-~10 $Å^{-1}$ and R range of 1-~3 Å was used for the fitting of CuO and Cu SAs/CN. The structure parameters such as the coordination number (*C. N.*), bond length (R), Debye-Waller factor (σ^2) and E_0 shift (ΔE_0) were fitted without anyone was fixed, constrained, or correlated.

Ascorbate peroxidase-like activity assay

The ascorbate peroxidase (APX)-like activity of the samples was verified in a scan mode by monitoring the absorption of ascorbate with H_2O_2 by UV-Vis spectroscopy at room temperature at 290 nm. Typically, experiments were carried out in buffer solution (pH range from 3 to 11) with a total volume of 2 mL containing Cu SAs/CN (25 μg mL^{-1}) and AA (0.0625 mM). Immediately after, H_2O_2 (5 mM) was added into the above mixture solution, the absorbance was collected over time. Meanwhile, the steady-state kinetic analysis was carried out at room temperature by varying the concentrations of AsA (0-0.125 mM) at fixed initial concentrations of Cu SAs/CN (25 μg mL^{-1}) and H_2O_2 (5 mM) in PBS solution or *vice versa*.

The initial reaction rate (V_0) against substrates was calculated based on the Michaelis-Menton function[3]:

$$\boldsymbol{v_0} = V_{max} \times [S]/(K_m+[S]) \quad (1)$$

where v_0 represents the initial reaction velocity, V_{max} refers to the maximal reaction velocity, K_m and $[S]$ are the Michaelis constant and concentration of substrate, respectively. Mean values of the initial rates of three traces were employed in the calculations. The apparent kinetic parameters (K_m and V_{max}) were further determined from the linear double-reciprocal plots[4]:

$$\mathbf{1}/\boldsymbol{v_0} = K_m \times [S]/V_{max} + \mathbf{1}/V_{max} \quad (2)$$

The catalytic rate constant (K_{cat}) of the samples were defined as:

$$K_{cat} = V_{max}/[E]$$

(3)

where [E] represents the molar concentration of Cu in Cu SAs/CN. Besides, the APX-like specific activity (SA) of Cu SAs/CN was calculated by:

$$\boldsymbol{a}_{\text{nanozyme}} = \boldsymbol{b}_{\text{nanozyme}}/[m]$$

(4)

$$\boldsymbol{b}_{\text{nanozyme}} = \boldsymbol{V} \times (\Delta \boldsymbol{A}/\Delta \boldsymbol{t})/(\boldsymbol{\varepsilon} \times \boldsymbol{l})$$

(5)

where $a_{nanozyme}$, $b_{nanozyme}$ and [m] represent the SA expressed in units per milligram (U mg^{-1}) nanozymes, the catalytic activity of nanozymes expressed in units (U) and the weight of nanozymes (mg) of each assay, respectively. V refers to the total volume of the reaction solution (μL), ε is the molar absorption coefficient of AsA at 290 nm (2800 M cm^{-1})[5], l is the path length of light traveling in the cuvette (cm), $\Delta A/\Delta t$ is the initial velocity of change at 652 nm/290 nm (min^{-1}). The SA values of the Cu SAs/CN are the slope of the straight line of $b_{nanozyme}$ against [m].

Recycling and selective tests of Cu SAs/CN as APX mimics

To explore the robustness and stability of Cu SAs/CN as APX mimics, the recycling tests of APX-like performance have been conducted. Experiments were carried out at room temperature in PBS buffer (10 mM, pH=7.4) with a total volume of 4 mL containing Cu SAs/CN (25 μg mL^{-1}) and AA (0.0625 mM). Then H_2O_2 (5 mM) was added into the above mixture solution, the absorbance was collected in real-time. After each cycle, Cu SAs/CN was recovered by centrifugation and washed with

deionized water. After three successive cycles, the Cu SAs/CN after reaction (Cu SAs/CN-AR) was collected by freeze-drying. Meanwhile, XRD and XPS analyses were also performed to characterize the crystal and surface structure of Cu SAs/CN-AR.

To further investigate the selectivity of Cu SAs/CN towards AsA, relevant Vitamin complexes such as VB1, VB2, VB3, VB6 and the symbolic peroxidase reducing substrate TMB were applied to replace AsA (0.0625 mM), the experiment was carried out in PBS buffer (10 mM, pH=7.4) containing Cu SAs/CN (25 μg mL^{-1}) and the Vitamin complexes (50 mM) and TMB (5 mM). Then H_2O_2 (5 mM) was added into the above mixture solution and incubated for 10 min, the absorbance was collected.

KSCN poisoning experiments

The thiocyanate ions (SCN^-) are widely known to poison the metal-centered catalytic sites. To explore the nature of the active sites of the sing-atom catalysts, the influence of SCN^- is also taken into account by the previously reported works. The SCN^{-1} experiments have also been performed by using KSCN to poison the metal-N_x sites (SCN^{-1} can form a stable chelate complex with the metal cations)[6-7]. Different concentrations of KSCN were firstly incubated with Cu SAs/CN (25 μg mL^{-1}) for 10 min. Then PBS solution containing AsA (0.0625 mM) and H_2O_2 (5 mM) was mixed with the above solution. The absorbance was collected in real-time mode.

Electrochemical assays

The electrochemical measurements were performed on an electrochemical

workstation (MATLAB) in a three-electrode electrochemical cell. Cu SAs/CN powder was dispersed into the Nafion alcohol solution. The well-distributed solution was then coated on the glass carbon electrodes (GCE) with a radius of 3 mm and dried at 60 ºC (working electrode). The assays were carried in PBS (10 mM, pH = 7.4) with a saturated Ag/AgCl electrode and Pt electrode as reference and counter electrode, respectively. The cyclic voltammetry (CV) and the linear sweep voltammetry were performed at a scanning rate of 5 mV s^{-1}. The amperometric responses of the prepared working electrodes were recorded by successive addition of H_2O_2 into the PBS solution (containing 80 mM AsA or not) at a potential of -0.2 V (*versus* Ag/AgCl).

Fluorimetric monitoring the oxidation of AsA based on APX-like activity

First, a series of concentrations AsA and Cu SAs/CN (25 μg mL^{-1}) were added to the PBS buffer solution (10 mM, pH = 7.4). After incubation for 6 min at 40 ºC, the acetate buffer solution (0.2 M, pH = 5.5) and OPDA (2 mM) was injected and followed with another incubation at 30 ºC for 20 min. The fluorescence spectra were recorded by a spectrometer with an excitation wavelength of 350 nm.

In situ monitoring the oxidation of AsA by Raman scattering

Typically, AsA solution (1 M) was mixed with Cu Sas/CN dispersion (0.5 mg mL^{-1}) and H_2O_2 solution (5 mM) in PBS buffer solution. The Raman scattering signals were recorded directly from the above mixture solution by real-time detection on a Raman spectrometer instrument (inVia Qontor, RENISHAW) with an excitation wavelength of 532 nm.

Theoretical calculations

Spin polarization density functional theory calculations were performed using the CASTEP package. The electron-correlation energies were treated with the generalized gradient approximation (GGA) in the Perdew-Burke-Ernzehof (GGA-PBE). The adsorption and reduction reaction of H_2O_2 on pristine g-C_3N_4 and single Cu atom doped g-C_3N_4 containing 18 carbon atoms and 25 nitrogen atoms have been studied comparatively. The Ultrasoft Pseudopotentials were used to describe the ionic core electrons, and a plane-wave basis set with a cutoff energy of 300 eV was adopted to treat the valence electrons. A threshold of self-consistent-field energy convergence was 2×10^{-5} eV/atom, and the maximum force and displacement of the convergence criterion for the structural optimizations were set to 0.05 eV/Å and 0.001 Å, respectively. The Monkhorst-Pack *k*-point sampling was generated with a 2×2×1 grid since the supercell is large enough, and all the atoms were allowed to relax in the calculations.

The adsorption energy (E_{ads}) is calculated according to the formula

$$E_{\mathrm{ads}} = E_{\mathrm{X\text{-}CN}} - (E_{\mathrm{X}} + E_{\mathrm{CN}}) \quad (6)$$

in which $E_{\mathrm{X\text{-}CN}}$ is the calculated total energy of the system with various species adsorbed either on the pristine g-C_3N_4 or the single Cu atom doped g-C_3N_4; E_X represents the energy of isolated adsorbates which can be H_2O_2 molecule, oxygen atom, OH or H_2O molecule; and E_{CN} is the energy of the pristine g-C_3N_4 or the single Cu atom doped g-C_3N_4. A more negative E_{ads} in equation (6) implies that the adsorption is thermodynamically more favorable. For the co-adsorption modes, the co-adsorption energy (E_{ads}) is calculated according to the formula

$$E_{ads} = E_{X\text{-}Y\text{-}CN} - (E_X + E_Y + E_{CN}) \quad (7)$$

$E_{X\text{-}Y\text{-}CN}$ is the calculated total energy of the system with two species co-adsorbed either on the pristine g-C_3N_4 or the single Cu atom doped g-C_3N_4 and E_Y means another co-adsorbed species. The reaction energy (ΔE) and the barrier (E_a) were defined as ΔE=E_{FS}-E_{IS} and ΔE=E_{TS}-E_{IS}, where TS, FS and IS are the transition state, final state and initial state.

Cell culture and viability assay

HeLa cells were provided by the Department of Laboratory Medicine, Southern Medical University. Herein, HeLa cells were cultured in Dulbecco's modified eagle medium (DMEM). All mediums contained 10% fetal bovine serum (FBS, Invitrogen) and 100 U mL^{-1} penicillin and 100 mg mL^{-1} streptomycin. Cells were cultured in a standard incubator at 37 °C with an atmosphere of 21% O_2 and 5% CO_2. The cell viability was evaluated by MTT assay. The HeLa cells were seeded in 96-well plates at a density of 8000 cells per well overnight. Then, the medium was replaced with 200 uL fresh DMEM medium containing various concentrations of Cu SAs/CN and incubated at 37 °C. Concerned about the low level of exogenous AsA and unspontaneous accumulation in HeLa cells in culture[8], incubating the HeLa cells with exogenous AsA ahead of time will benefit the enrichment of AsA, reaccumulation of the endogenous AsA and in favor of experimental observation. Therefore, for H_2O_2 treatment, the cells were pre-treated with low dosage of Cu SAs/CN (25 μg/mL) and AsA (0.0625 mM) for 24 h, and then incubated with or without 500 μM H_2O_2. After 24 h, the medium was replaced with a 10% MTT solution. Then 100 μL of DMSO

was added, and the plates were analyzed with a microplate reader (Thermo Scientific VL0L0TD0) with an absorbance at 575 nm. Each experiment was performed at least three times.

Cellular uptake of Cu SAs/CN

Cellular internalization of Cu SAs/CN was determined by a confocal laser scanning microscope (CLSM, FVf3000, Olympus). HeLa cells were seeded on a confocal dish overnight. Upon being treated with Cu SAs/CN (25 μg/mL), the cells were incubated for 24 h. Then the cells were washed three times with PBS buffer. And the intracellular fluorescence level of the cells was detected with CLSM with an excitation wavelength of 405 nm and an emission filter of 410-510 nm.

Serum stability test

For serum stability, Cu SAs/CN was incubated in DMEM cell culture medium supplemented with 10% FBS, and the hydration size of Cu SAs/CN was measured at different times, respectively.

Detection of intracellular reactive oxygen species (ROS) production

The generation of the intracellular ROS was detected by the DCFH-DA assay through a confocal laser by the microscope (FV3000, Olympus). HeLa cells were seeded on a Confocal Dish overnight. Upon being treated with 25 μg/mL Cu SAs/CN and 0.0625 mM AsA, the cells were incubated for 24 h. Then 500 μM H_2O_2 was added and incubated for 1 h. For the DCFH-DA assay, the cells were then washed three times with PBS buffer and incubated with 10 μM DCFH-DA in an FBS-free DMEM medium for 30 min in the dark. The cells were further washed three times with PBS

buffer, and the intracellular ROS level was detected with a confocal microscope with an excitation wavelength at 488 nm.

Statistical analysis

The experimental results for the TEM, SEM images are shown as raw data without preprocessing. The measured particle sizes were presented as their average values. The measurement for each assay was repeated in triplicate unless otherwise noted. Error bars in each Figure represent the standard deviation.

Table S1. Atomically percentage of CN and Cu SAs/CN samples determined by XPS.

Samples	C 1s (%)	N 1s (%)	O 1s (%)	Cu 2p (%)
CN	54.7	41.3	4.0	—
Cu SAs/CN-30	54.6	44.39	0.96	0.05
Cu SAs/CN-60	57.5	41.5	0.8	0.12
Cu SAs/CN-90	51.1	47.5	1.38	0.21

Table S2. EXAFS fitting parameters at the Cu K-edge for Cu foil, CuO and Cu SAs/CN (S_0^2= 0.79).

**Sample**	**Path**	***C.N.***	***R* (Å)**	**$\sigma^2 \times 10^3$ (Å^2)**	***ΔE* (eV)**	***R*-factor**
Cu foil	Cu-Cu	12*	2.52±0.01	8.4±0.3	3.8±0.5	0.002
	Cu-O	3.8±0.4	1.95±0.01	4.3±1.1	6.0±1.3	
CuO	Cu-Cu	6.7±2.8	2.96±0.01	11.9±5.6	8.8±3.2	0.006
	Cu-Cu	1.7±1.3	3.14±0.01	2.9±1.6		
Cu SAs/CN	Cu-N	4.6±1.0	1.94±0.02	12.2±3.0	3.5±2.2	0.016

[a]*C.N.*: coordination numbers; [b]*R*: bond distance; [c]σ^2: Debye-Waller factors; [d] Δ*E*: the inner potential correction. *R* factor: goodness of fit. The S_0^2 was set as 0.79, the experimental EXAFS fit of metal foil by fixing CN as the known crystallographic value.

Table S3. Comparison of the kinetic constants of Cu SAs/CN, natural APXs and APX mutants for AsA substrate.

Catalysts	[E/Cu] (M)	K_m (mM)	V_{max} (10^{-8} M s^{-1})	k_{cat} (s^{-1})	k_{cat}/K_m($mM^{-1}s^{-1}$)	Ref.
Cu SAs/CN	$1.2*10^{-9}$	0.01	19.16	$1.60*10^{2}$	$1.60*10^{4}$	This work
GST-APXa	—	4	15	—	—	9
GST-APXb	—	1	20	—	—	
APX from *Chlorella vulgaris*	—	0.11	—	$6.96*10^{2}$	$6.27*10^{3}$	10
APX from *Musa paradisaca* leaf juice	—	0.13	—	40.42	$3.1*10^{2}$	11
Recombinant APX-B 37	—	0.22	—	$3.4*10^{3}$	$1.60*10^{4}$	12
Recombinant APX-B 25	—	0.12	—	$2.2*10^{3}$	$1.84*10^{4}$	
Native APX-B 25	—	0.17	—	—	—	
Wild type	—	—	—	33.10	—	13

APX1M	—	—	—	12.20	—	
APX3M	—	—	—	8.36	—	
APX3M/W179F	—	—	—	3.10	—	
APX3M/R172N	—	—	—	0.22	—	
rpAPX	—	0.35	—	$1.59*10^2$	$4.48*10^2$	14
$S160M_R$	—	0.10	—	$1.56*10^2$	$1.53*10^3$	

[E/Cu] refers to the molar concentration of the Cu active sites on Cu SAs/CN.

Table S4. Comparison of the kinetic constants of Cu SAs/CN, natural APXs and APX mutants for H_2O_2 substrate.

Catalysts	[E/Cu] (M)	K_m (mM)	V_{max} (10^{-8} M s^{-1})	k_{cat} (s^{-1})	k_{cat}/ K_m($mM^{-1}s^{-1}$)	Ref.
Cu SAs/CN	$1.2*10^{-9}$	0.45	17.72	$1.48*10^2$	$3.28*10^2$	This work
GST-APXa	—	0.30	1	—	—	9
GST-APXb	—	0.70	3	—	—	
APX from *Chlorella vulgaris*	—	0.02	—	—	—	10
APX from *Musa paradisaca* leaf juice	—	0.23	—	27.24	$1.18*10^2$	11
Recombinant APX-B 37	—	0.08	—	—	—	
Recombinant APX-B 25	—	0.04	—	—	—	12
Native APX-B 25	—	0.03	—	—	—	

[E/Cu] refers to the molar concentration of the Cu active sites on Cu SAs/CN.

Table S5. Comparison of the specific activity between Cu SAs/CN and natural APXs.

Samples	Specific activity (U mg^{-1})	Ref.
Cu SAs/CN	461.60	This work
APX from *Musa paradisaca* leaf juice	203.04	11
APX form *Rhizophagus manihotis*-plants	305	15
S. polyrhizal extract	0.23	16
S. polyrhizal extract after purification	1.44	
APX from *Chlorella vulgaris*	3.32	10
APX from *Chlorella vulgaris* purified by Superdex 200	918	
APX from *Chlorella vulgaris* purified by phenyl superose	1307	

Table S6. The band assignments of the Raman bands of AsA.

W_{Raman} (cm^{-1})	Band assignments
823.3	C-C-O symmetric stretching mode
1692.6	C=C stretching mode

Figure S1. Morphology characterization of Cu SAs/CN. (a) SEM image, (b) HAADF-STEM image and corresponding elemental mapping (b). (c) EDS image of Cu SAs/CN.

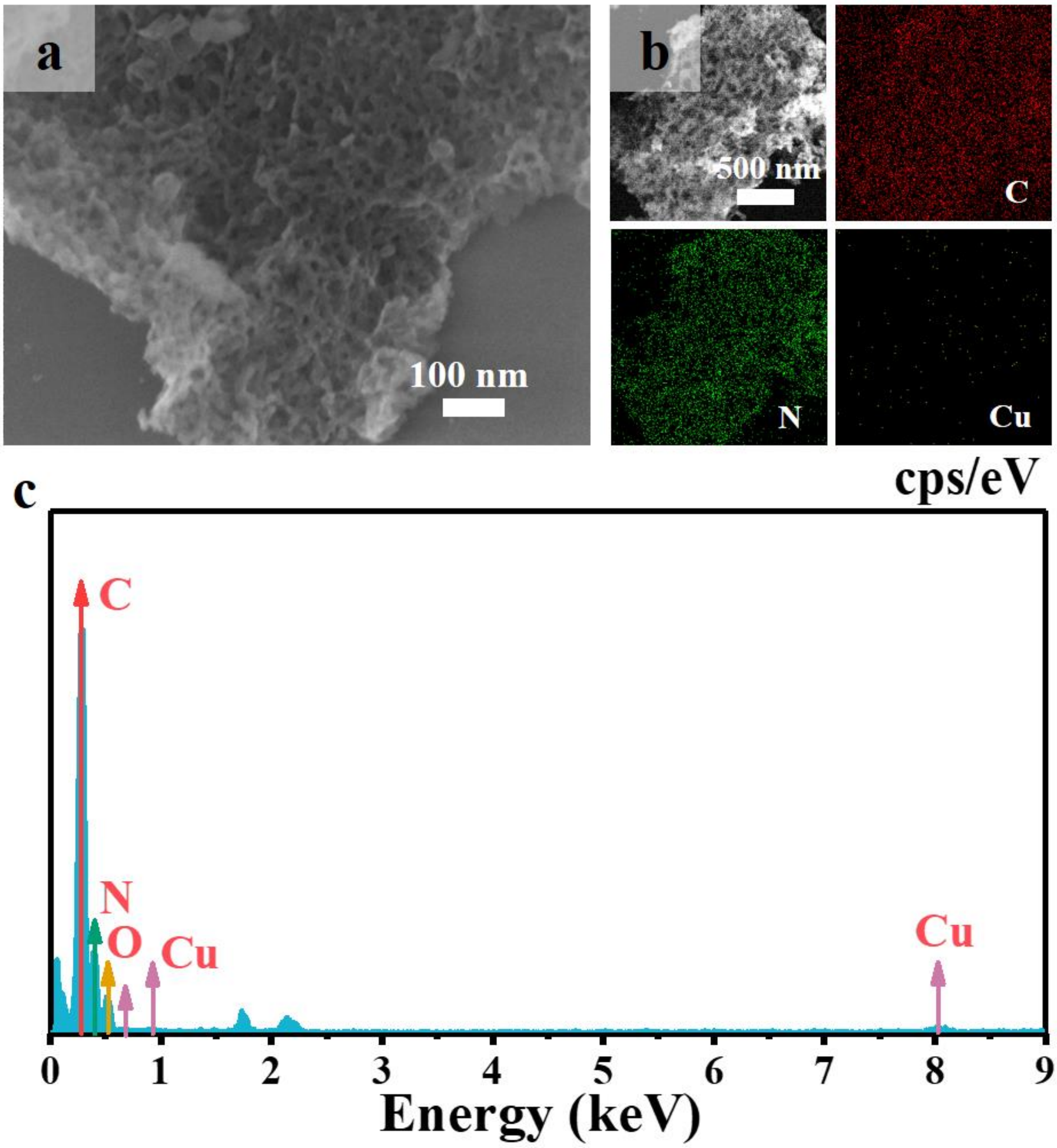

Figure S2. Morphology characterization of CN. (a) TEM image and corresponding elemental mapping. AC HAADF-STEM images, (b) dark-field, (c) bright-field.

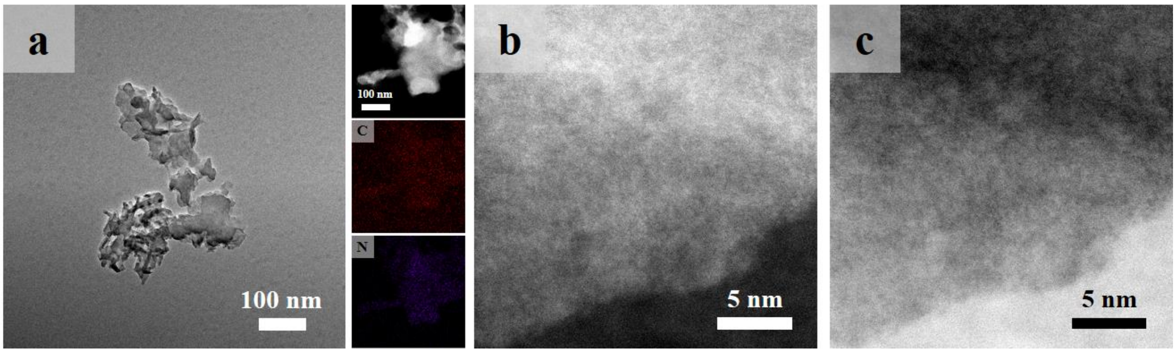

TEM image and EDS mapping result in panel (a) suggest that CN is fabricated with an ultrathin porous layer structure, C and N elements are uniformly dispersed on CN structure. Meanwhile, from the AC HAADF-STEM images in panels (b) and (c), no bright spots (dark-field) or dark spots are founded, demonstrating that the CN matric is prepared with a homogeneous structure.

Figure S3. XRD patterns of as-prepared samples.

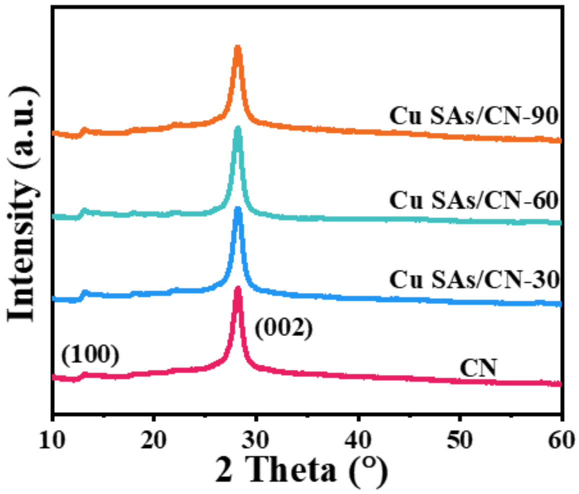

Figure S4. AC HAADF-STEM images of Cu SAs/CN, (a) dark-field, (b) bright-field. Statistical size distribution of 100 bright dots in the AC-HAADF-STEM image in Figure 1b.

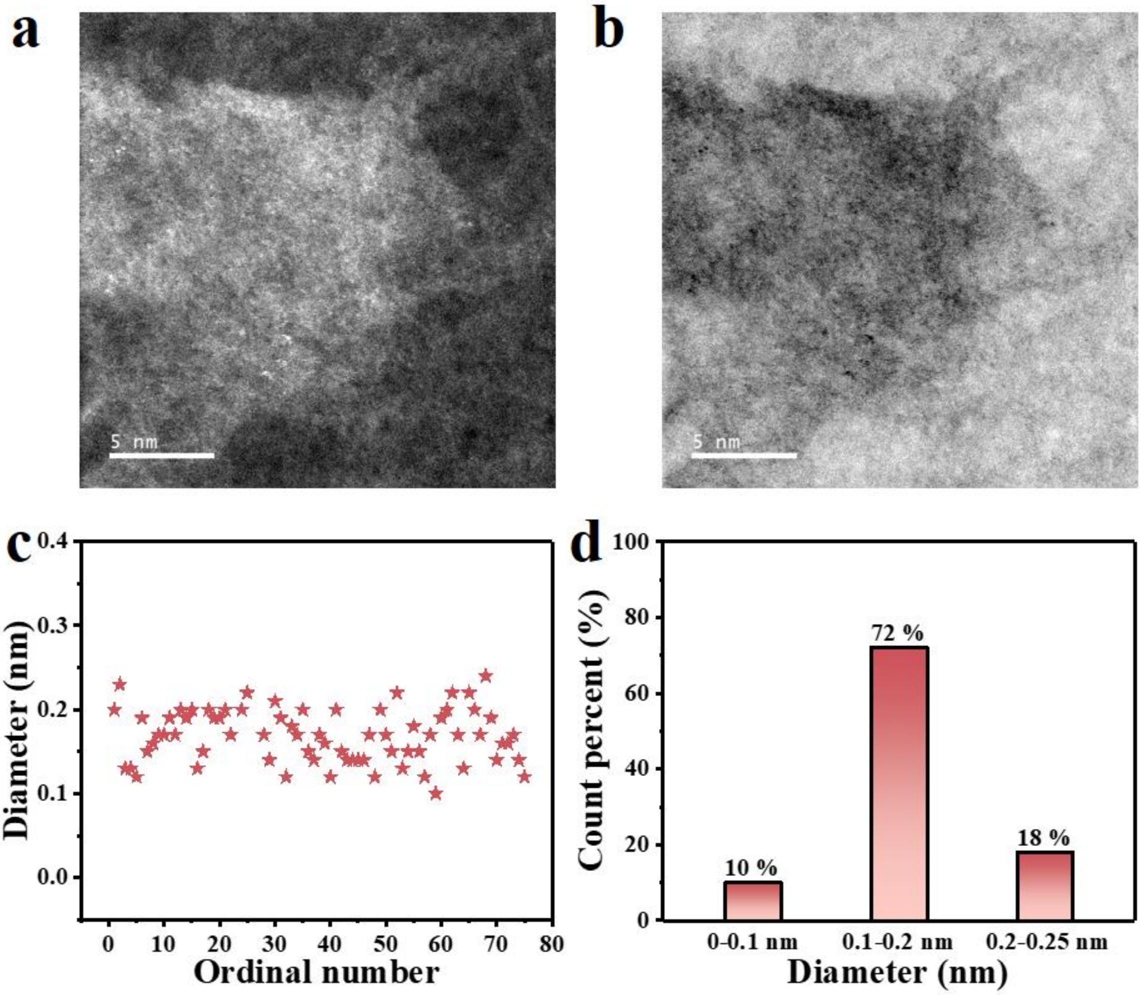

Figure S5. FTIR patterns of as-prepared samples.

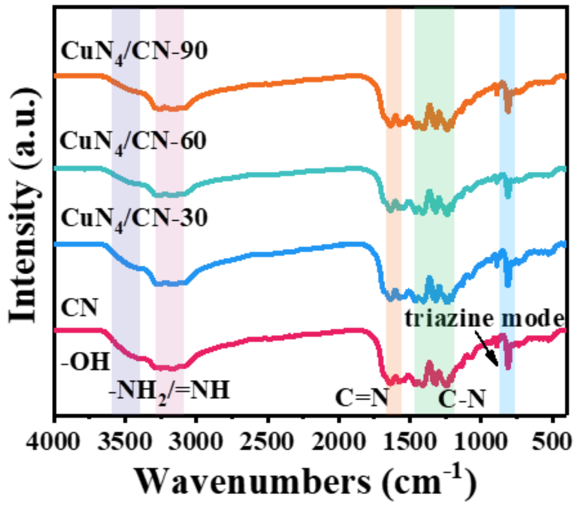

The broad peaks in the range of 2900 to 3648 cm^{-1} can be ascribed as the -OH and uncondensed terminal amino group $-NH_2$ or =NH groups. Meanwhile, the representative stretching modes C=N and the in-plane =C-N can be observed in 1100-1750 cm^{-1}[17-18].

Figure S6. BET data of as-prepared samples. (a) N_2 adsorption and desorption isotherms. (b) The corresponding pore size distribution.

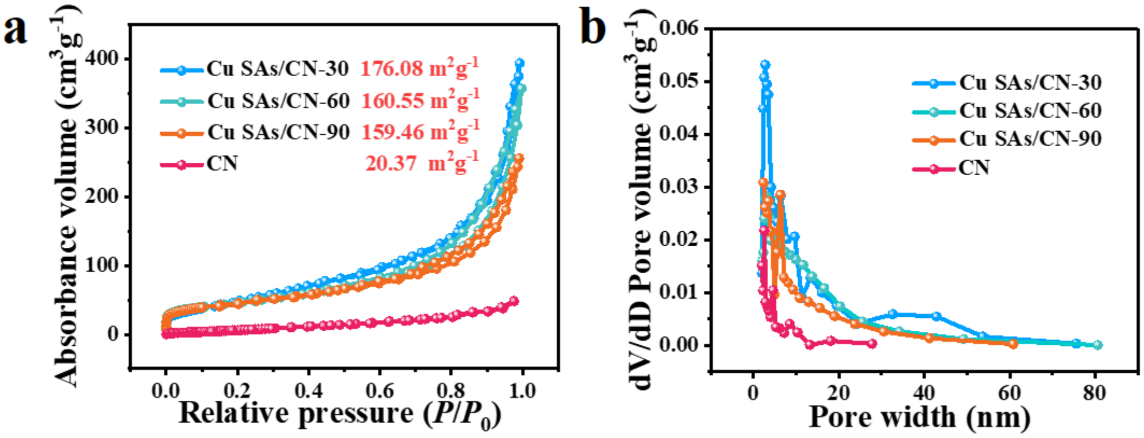

The porous structure endows Cu SAs/CN samples with high specific surface area and abundant hierarchical nanopores. Cu SAs/CN (30, 60, 90) samples present a high adsorption capacity at high relative pressures (P/P_0>0.8), featuring BET surface areas of 176.08, 160.55 and 159.46 m^{2}g^{-1} when compared with the CN matrix (20.37 m^{2}g^{-1}). The BET surface area of Cu SAs/CN sample are 8.64, 7.88 and 7.83 times higher than that of CN. After the electrochemical deposition treatment with increasing time, the specific surface area and pore size distribution exhibit lightly decline, which may contribute to the encapsulating of Cu single atoms.

Figure S7. XPS spectra of the as-prepared samples. The survey spectra (a) and high-solution XPS spectra of C 1s (b), N 1s (c) and Cu 2p (d).

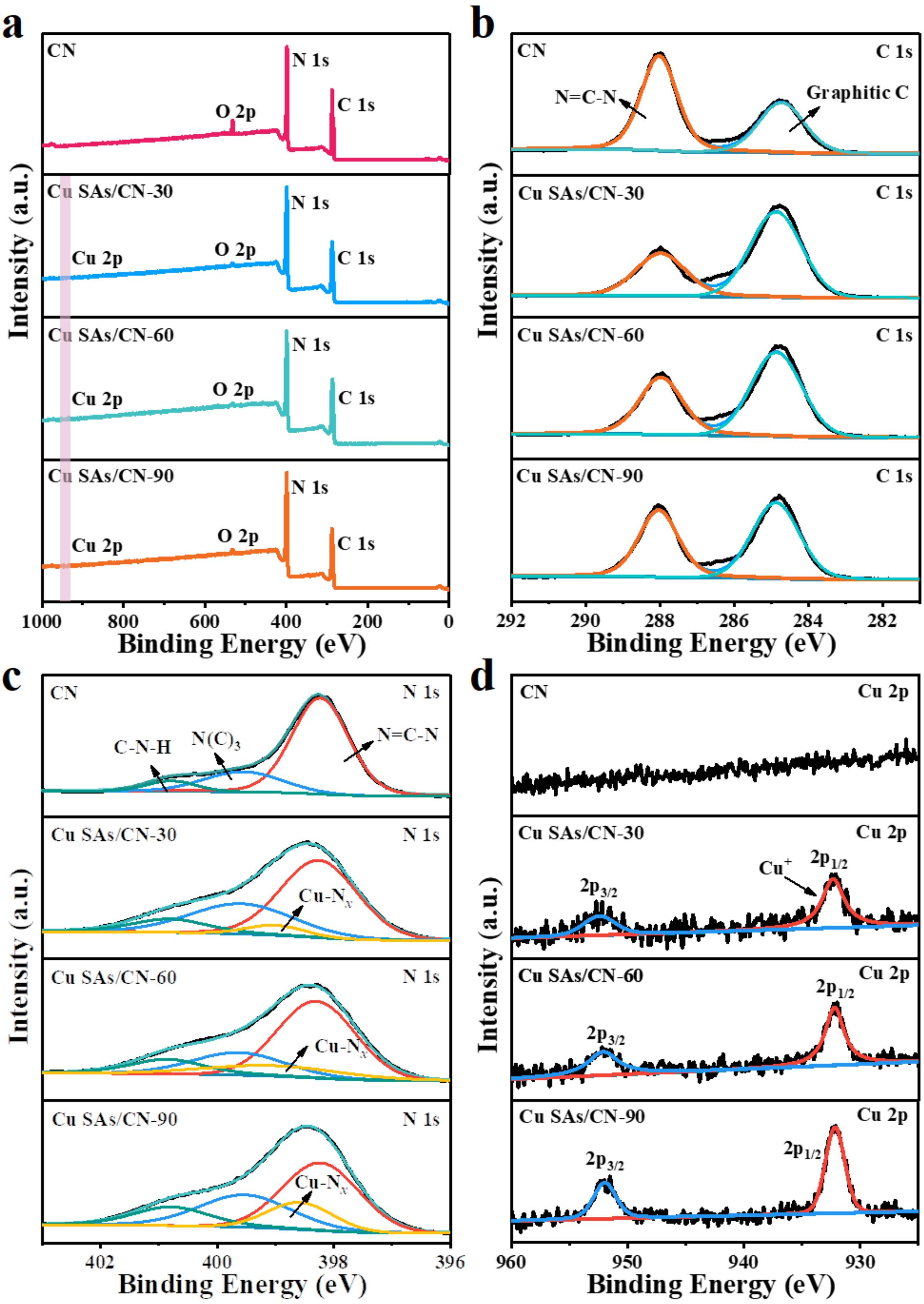

The high-solution C 1s in panel (b) featured two peaks with the binding energy of 284.7 and 288.0 eV, which can be ascribed to the sp^2 C-C bonds and sp^2 bonded carbon from the N-containing aromatic rings N-C=N.

Figure S8. Fitting curves of the EXAFS of Cu SAs/CN in the k-space.

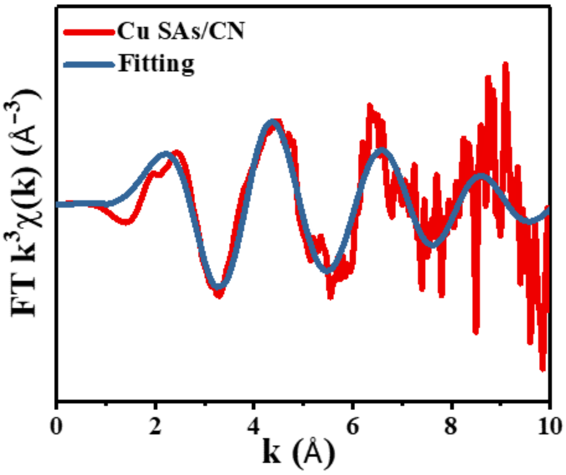

Figure S9. Fitting curves of the EXAFS of Cu foil and CuO in the R-space and k-space, respectively. (a), (b) Cu foil, (c), (d) CuO.

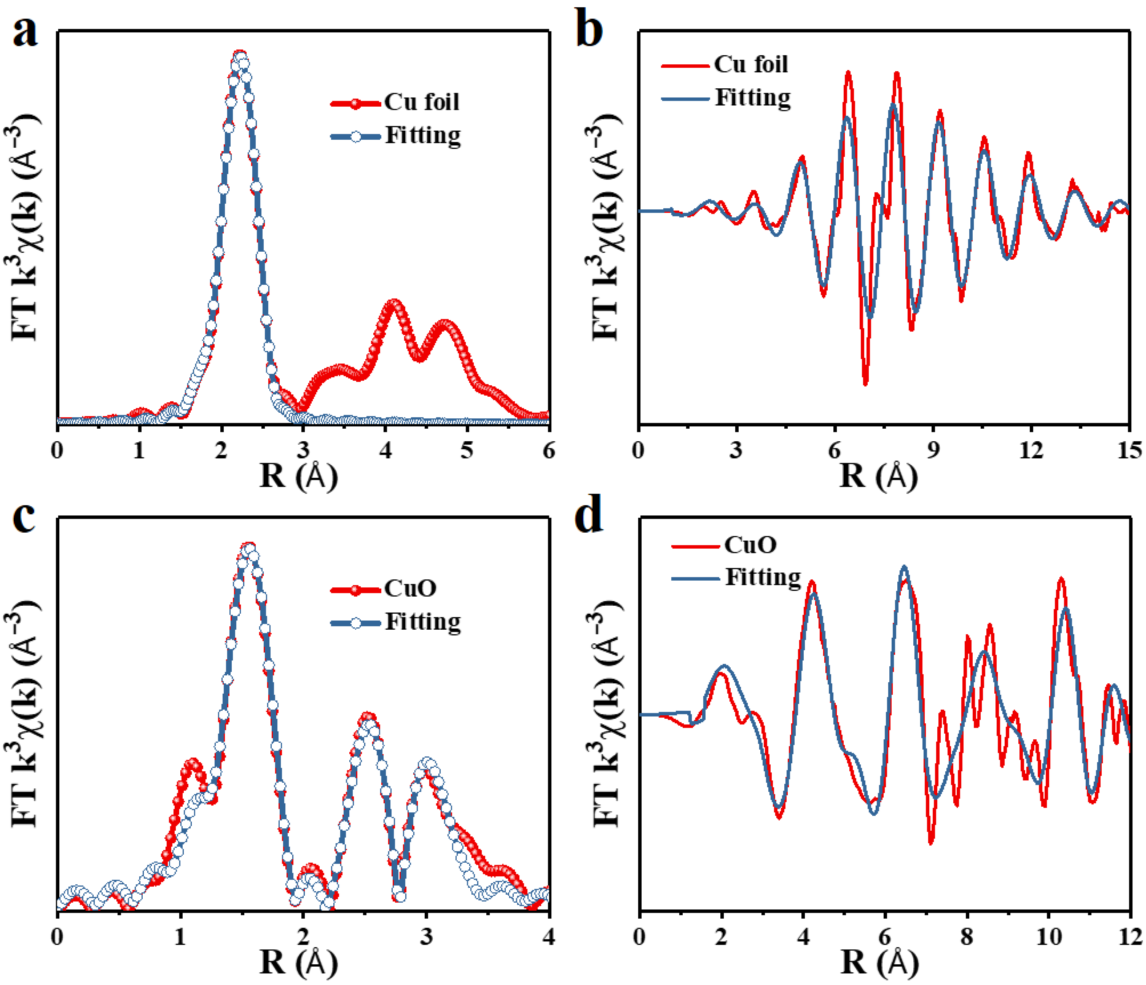

Figure S10. The electronic structure of CN is calculated by DFT. (a) The optimized unit structure (top view and side view). (b) Total density states and orbital distribution of each element. (c) Projected density states image.

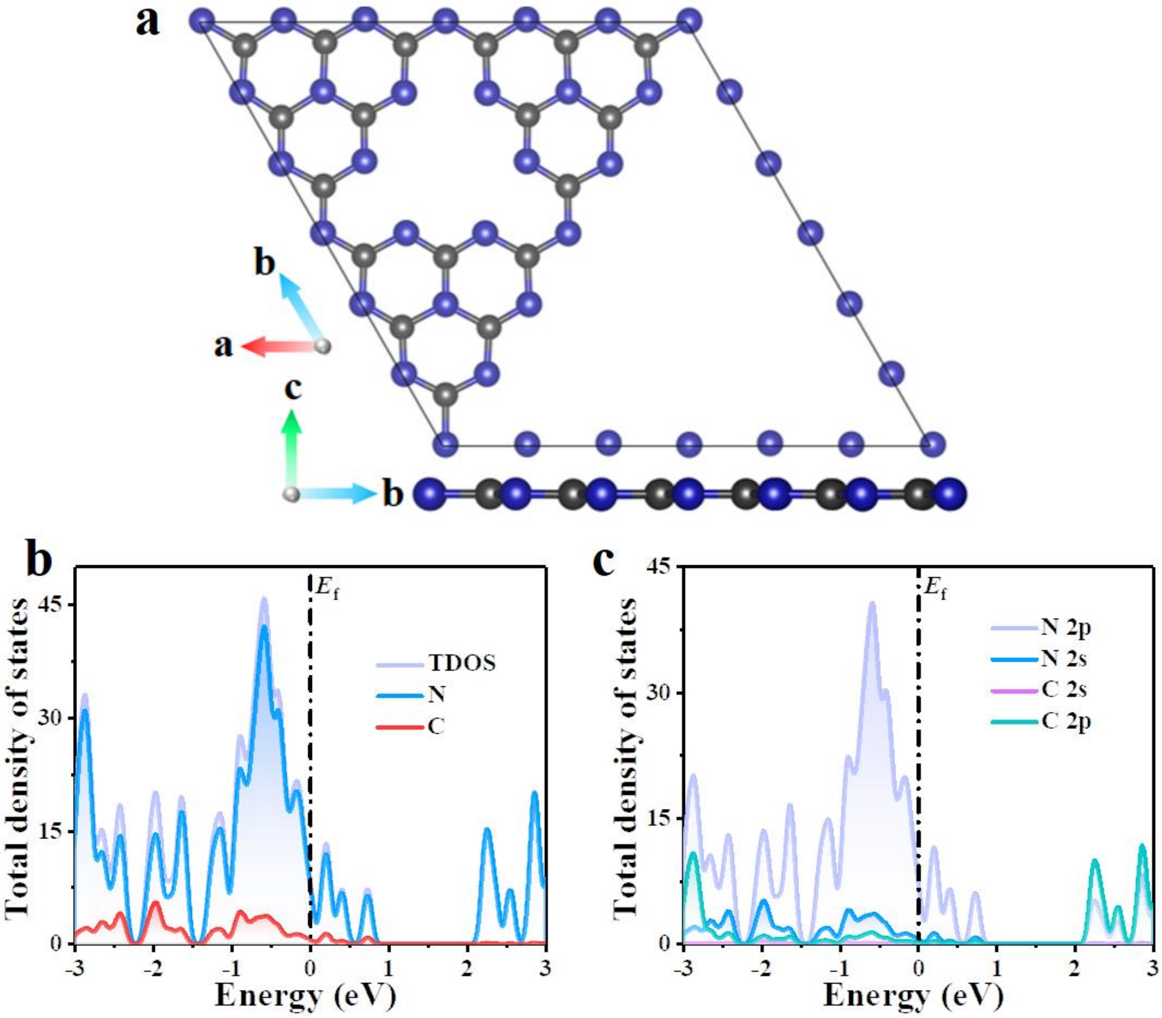

Figure S11. The differential charge distribution maps of CN (a) and Cu SAs/CN (b). The blue area refers to the charge loss, the red area refers to the charge accumulation. The calculated Hirshfeld charge analysis of N atoms and Cu atoms in CN (c) and (d). The negative and positive values represent the electron loss and accumulation, respectively. The following table shows the Hirshfeld charge and Mulliken charge of CN and Cu SAs/CN.

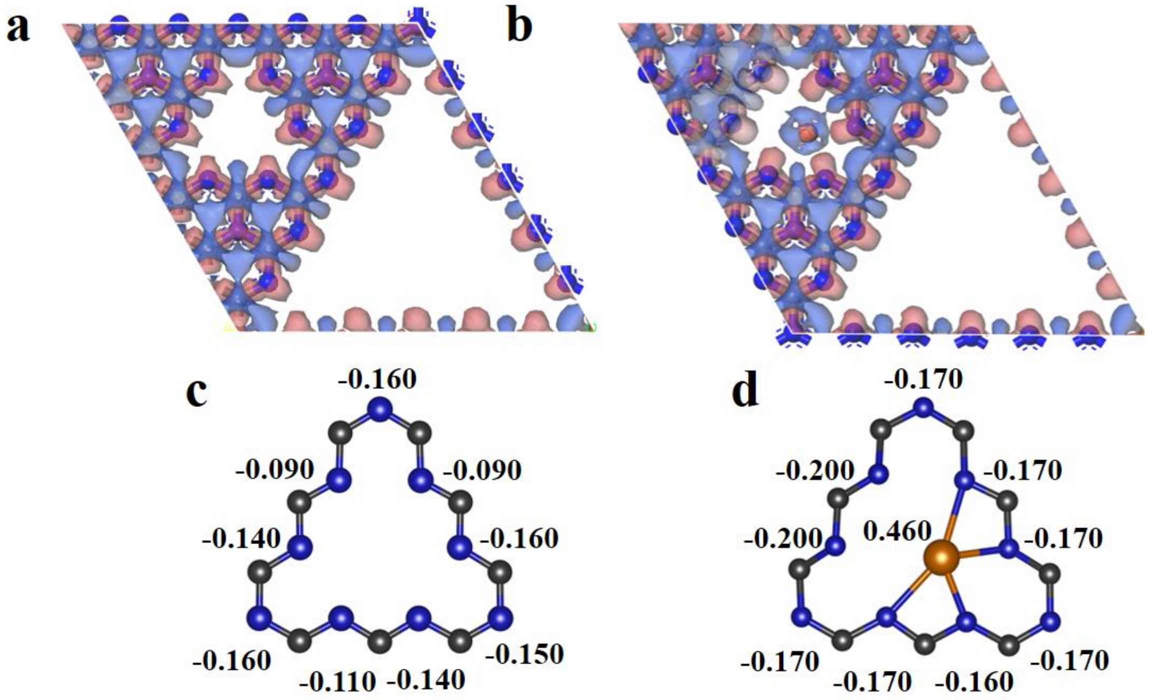

The more negative values from the Hirshfeld and Mulliken charge calculations in the following table suggest that the electrons would pass through the single Cu atoms to transfer into the coordinated N atoms. Both of the differential charge distribution maps, the Hirshfeld charge or the Mulliken charge calculations present an electron transfer process between the anchored Cu atoms and the coordinated N atoms.

Samples	Hirshfeld charge (electrons) / Mulliken charge (electrons)				
	N_1	N_2	N_3	N_4	N_5
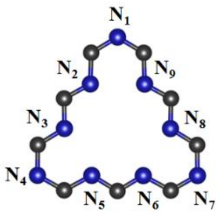	-0.160	-0.090	-0.140	-0.160	-0.110
	-0.400	-0.340	-0.350	-0.350	-0.340
	N_6	N_7	N_8	N_9	Cu
CN	-0.140	-0.150	-0.160	-0.090	—
	-0.360	-0.400	-0.360	-0.340	—
	N_1	N_2	N_3	N_4	N_5
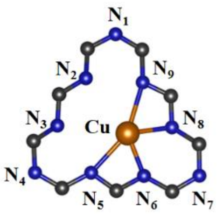	-0.170	-0.200	-0.200	-0.170	-0.170
	-0.370	-0.450	-0.450	-0.450	-0.480
	N_6	N_7	N_8	N_9	Cu
Cu SAs/CN	-0.160	-0.170	-0.170	-0.170	0.460
	-0.510	-0.370	-0.520	-0.480	1.200

Figure S12. Electrochemical measurements of Cu SAs/CN and CN modified glass carbon electrodes. (a) Electrochemical impedance spectra (EIS). (b), (c) The Mott-Schottky plots of CN and Cu SAs/CN, respectively. (d) Polarization curves.

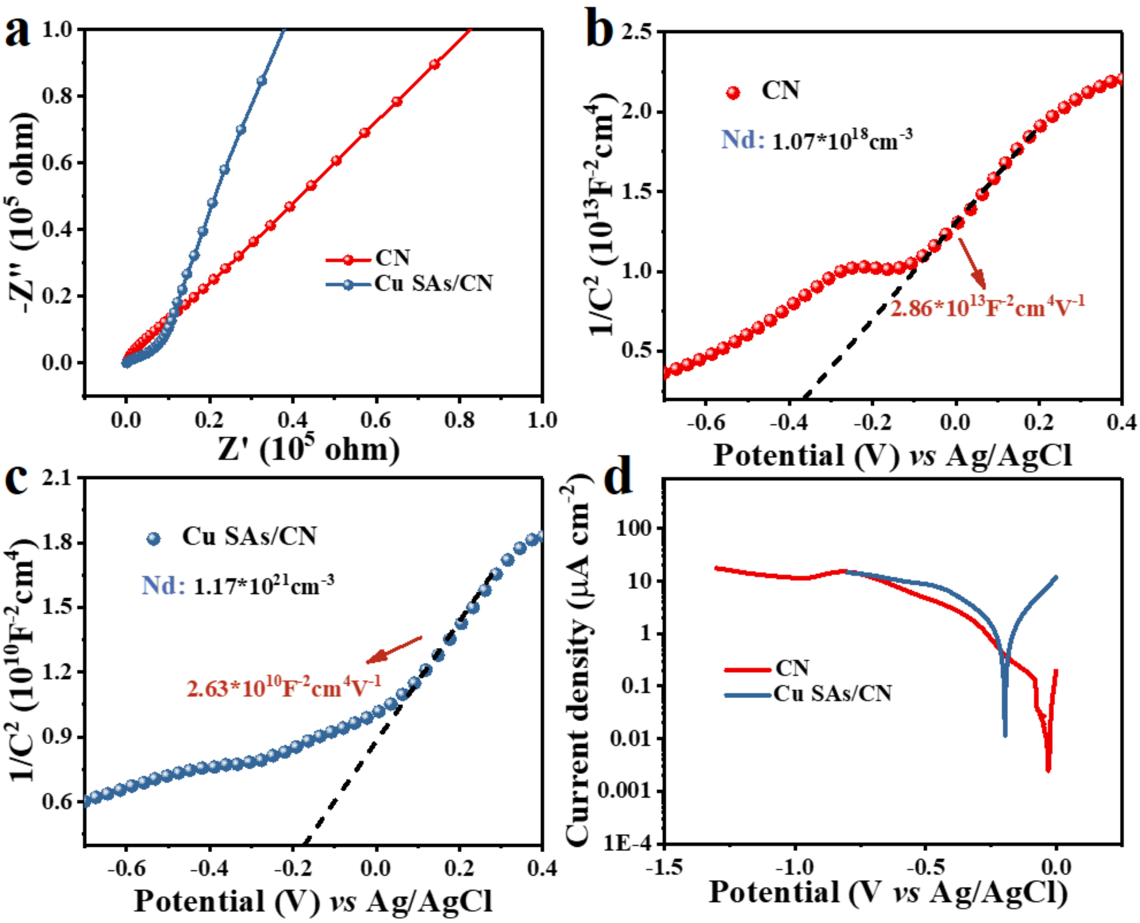

As depicted in panel (a), a smaller medium frequency semicircle is observed in the EIS Nyquist plots, revealing the more efficient electron charge transfer in Cu SAs/CN. The carrier densities are then determined by Mott-Schottky measurement, according to the equation:

$$N_d = 2/(\varepsilon_0 \varepsilon e_0)[d/(1/C^2)/dV]^{-1}$$

Where N_d, ε_0, and e_0 represent the donor density, the permittivity of vacuum and electron charge. ε is the dielectric constants of CN (4.6)[19]. Cu SAs/CN exhibits a smaller slope of Mott-Schottky (n-type), demonstrating a larger charge density. The

carrier density is further calculated to be $1.17*10^{21}$ cm^{-3} for Cu SAs/CN, almost 10^3 times higher than CN ($1.01*10^{18}$ cm^{-3}). The faster charge transfer may allow more available charges for driving the catalysis reactions[20]. The polarization curves in panel (d) also support the above-proposed idea. A more negative equilibrium potential and higher current density demonstrate that Cu SAs/CN is more desirable to react with substrates than CN.

Figure S13. Ultraviolet-visible (UV-vis) absorption spectra of different systems. (a) APX-like assay on Cu SAs/CN. (b) Comparison of the APX-like performance of Cu SAs/CN and CN.

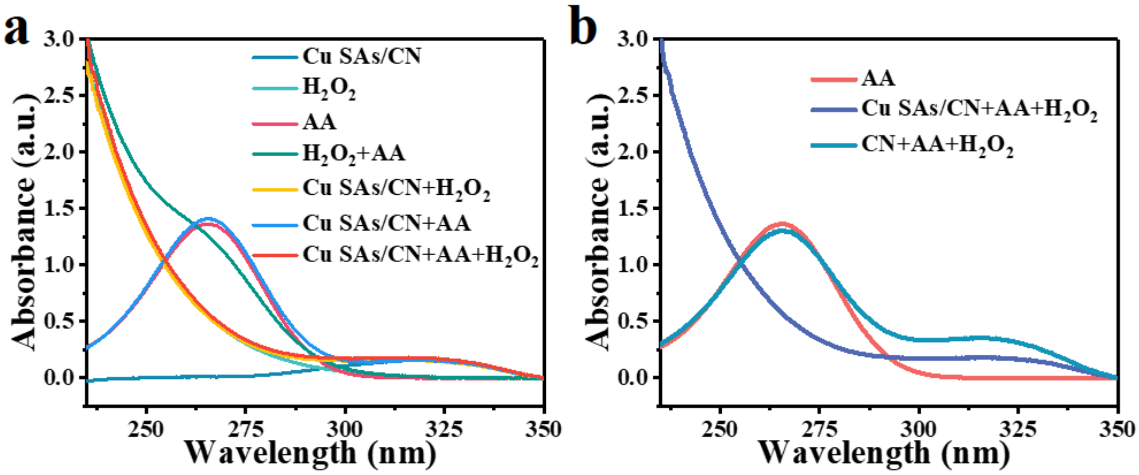

Figure S14. UV-vis absorption spectra in a real-time mode of the AsA at 290 nm catalyzed by Cu SAs/CN before and after SCN^{-1} poisoning.

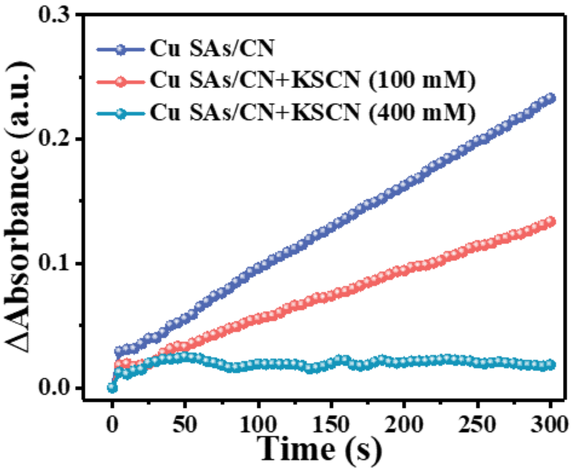

Figure S15. The effect of pH and temperature on the APX-like activity of Cu SAs/CN. (a) pH, (b) the APX-like activity of Cu SAs/CN and the control system (water) under different temperatures, (c) the relative APX-like activity of Cu SAs/CN, where the self-decomposition of AsA in the presence of H_2O_2 under an increasing temperature was deleted.

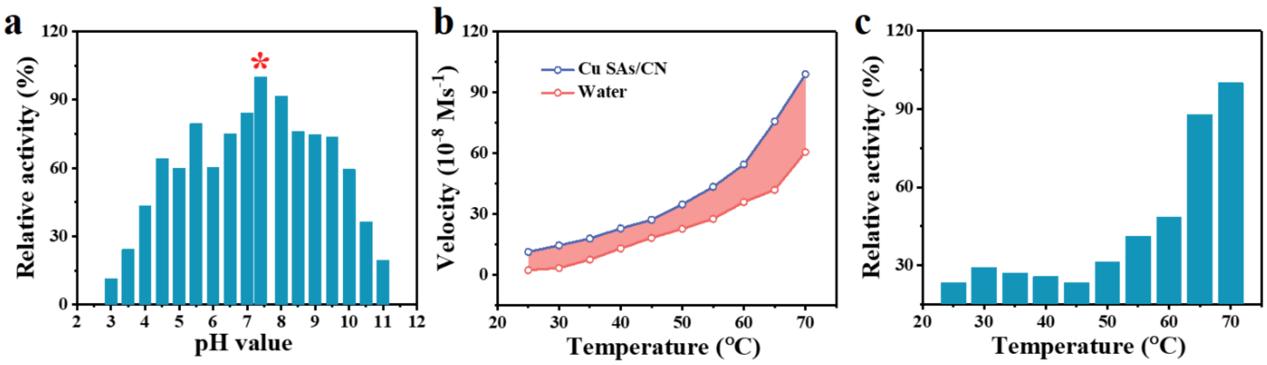

the APX mimetic activity of Cu SAs/CN exhibits a pH- (range from 3 to 11) and temperature-dependent (range from 25 to 70 ºC) behavior. The optimal pH was approximately around 7.4 in panel (a). Besides, the APX-like performance of Cu SAs/CN is improved with the increasing temperature in panel (b-c). Whereas the control system (AsA-H_2O_2) was facilely self-decomposed above 45 ºC. Therefore, to avoid the interference of the self-decomposition of AsA under high temperature, we choose the room temperature (25 ºC) throughout the APX-like assay.

Figure S16. Steady-state kinetic assay of Cu SAs/CN. The double-reciprocal plot corresponds to Figure 2b.

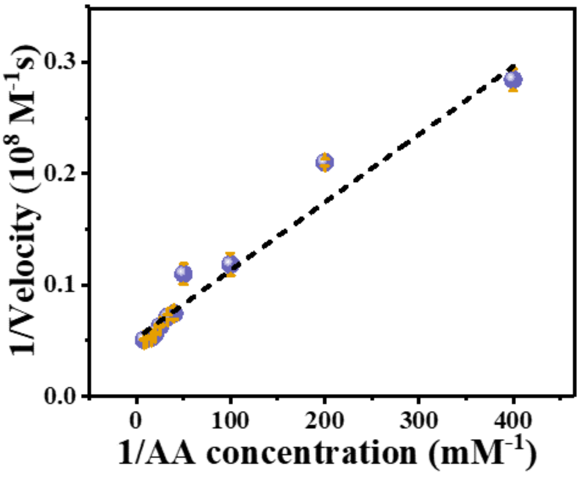

Figure S17. Steady-state kinetic assay of Cu SAs/CN. (a) Michaelis-Menton curves by varying H_2O_2 concentration at a constant concentration of AsA. (b) The corresponding double-reciprocal plot.

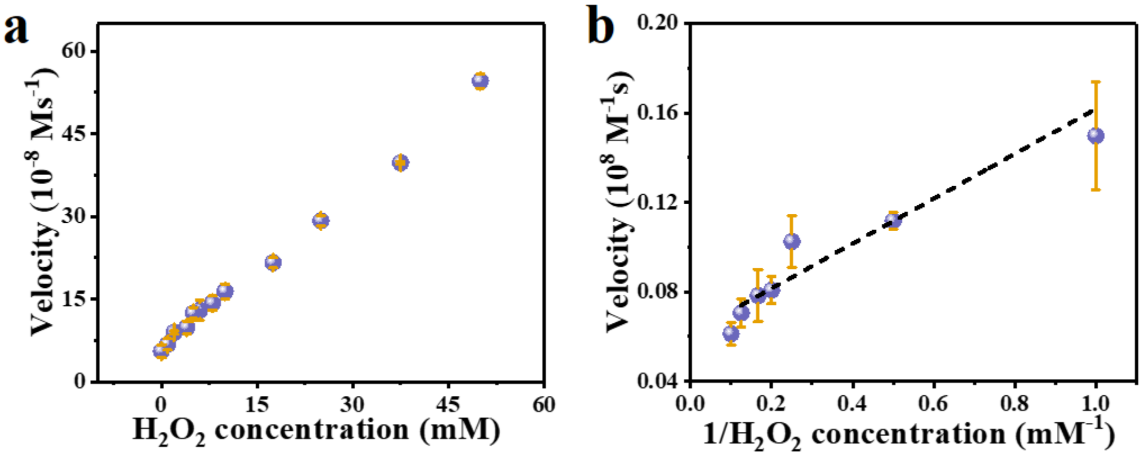

Figure S18. Double-reciprocal plots of Cu SAs/CN as APX mimics at a fixed concentration of one substrate versus concentrations of the second one. (a) H_2O_2, (b) AsA.

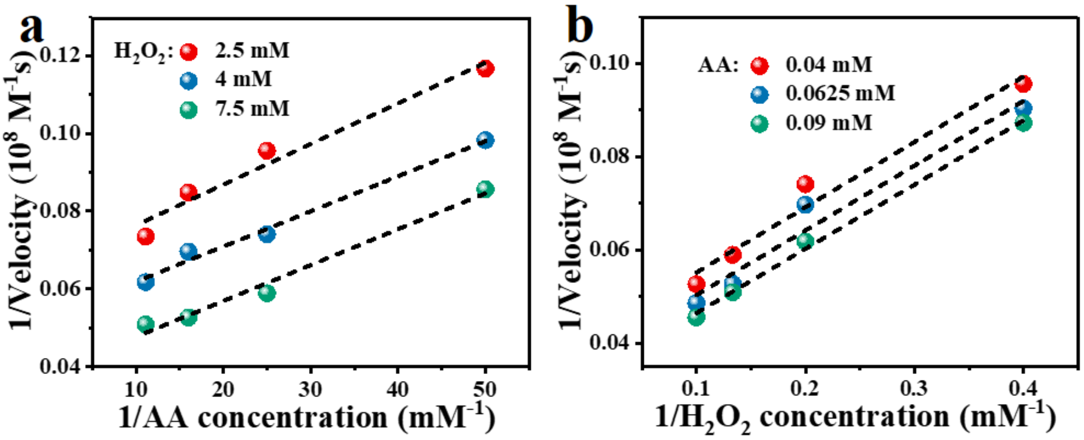

The obtained curves in an approximately linear manner match the ping-pong mechanism where the enzyme bind with the first substrate, releasing the product before reacting with the second one. This is in good agreement with the natural APX.

Figure S19. Stability and recyclability of Cu SAs/CN as APX mimics. (a) The recycle tests of Cu SAs/CN as APX mimics. (b) The crystal structure of Cu SAs/CN was characterized by XRD analysis before and after the recycling tests. The surface structure of Cu SAs/CN examed by XPS analysis before and after the recycling tests. The survey spectra (c) and high-solution XPS spectra of C 1s (d), N 1s (e) and Cu 2p (f).

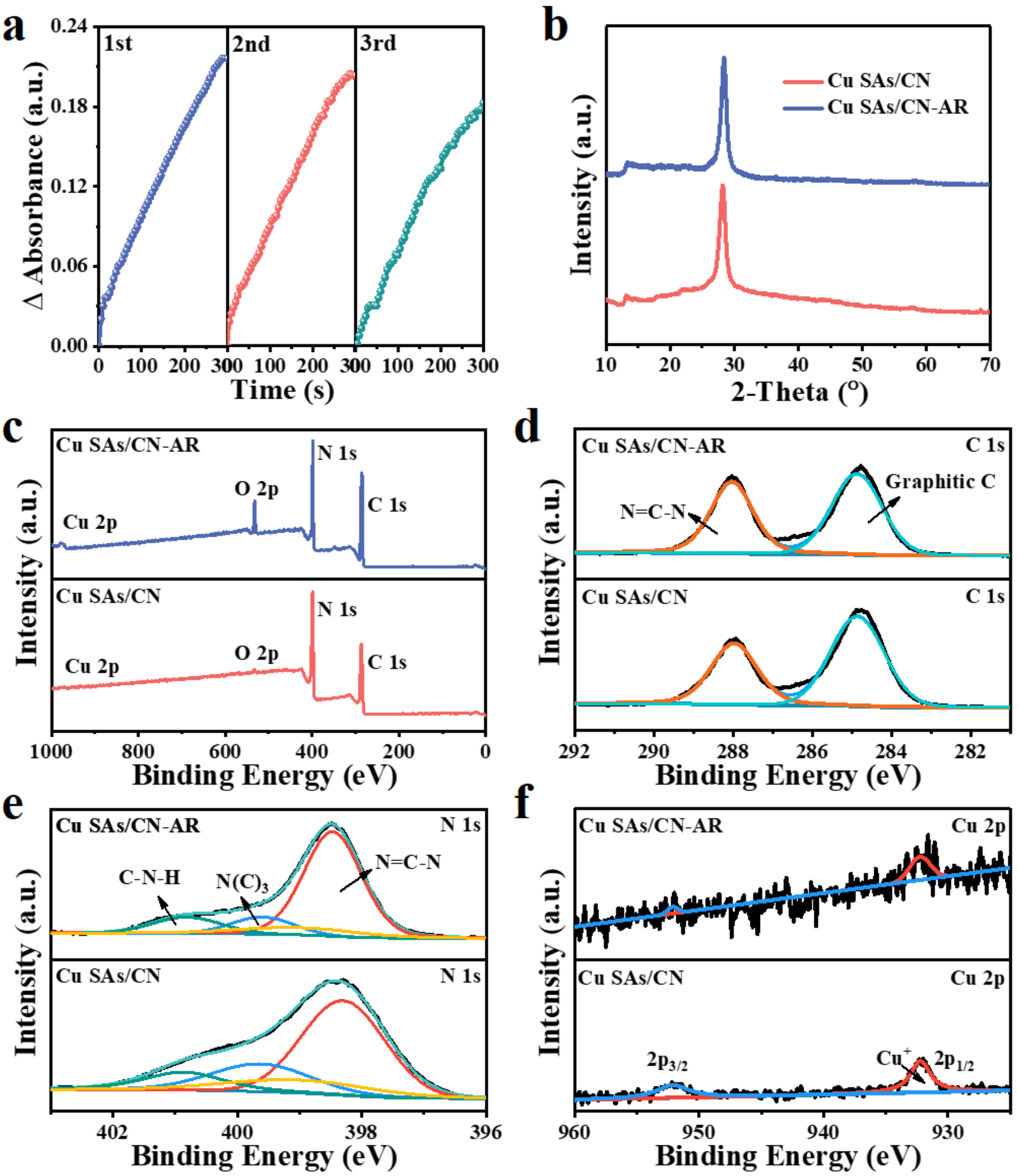

After each cycle, Cu SAs/CN was recovered by centrifugation and washed with deionized water. After three successive cycles, Cu SAs/CN suffered a little loss in APX-like performance. Nevertheless, Cu SAs/CN retained 80+% activity and the intact phase (XRD) and surface structure. The sufficient stability makes Cu SAs/CN more competitive as APX mimics.

Figure S20. Selectivity of Cu SAs/CN as APX mimics towards AsA. The relevant vitamin complexes (a)-(d): VB1, VB2, VB3 and VB6, respectively. (e) The typical peroxidase reducing substrate TMB. (f) The effect of pH on the peroxidase-like activity of Cu SAs/CN towards TMB.

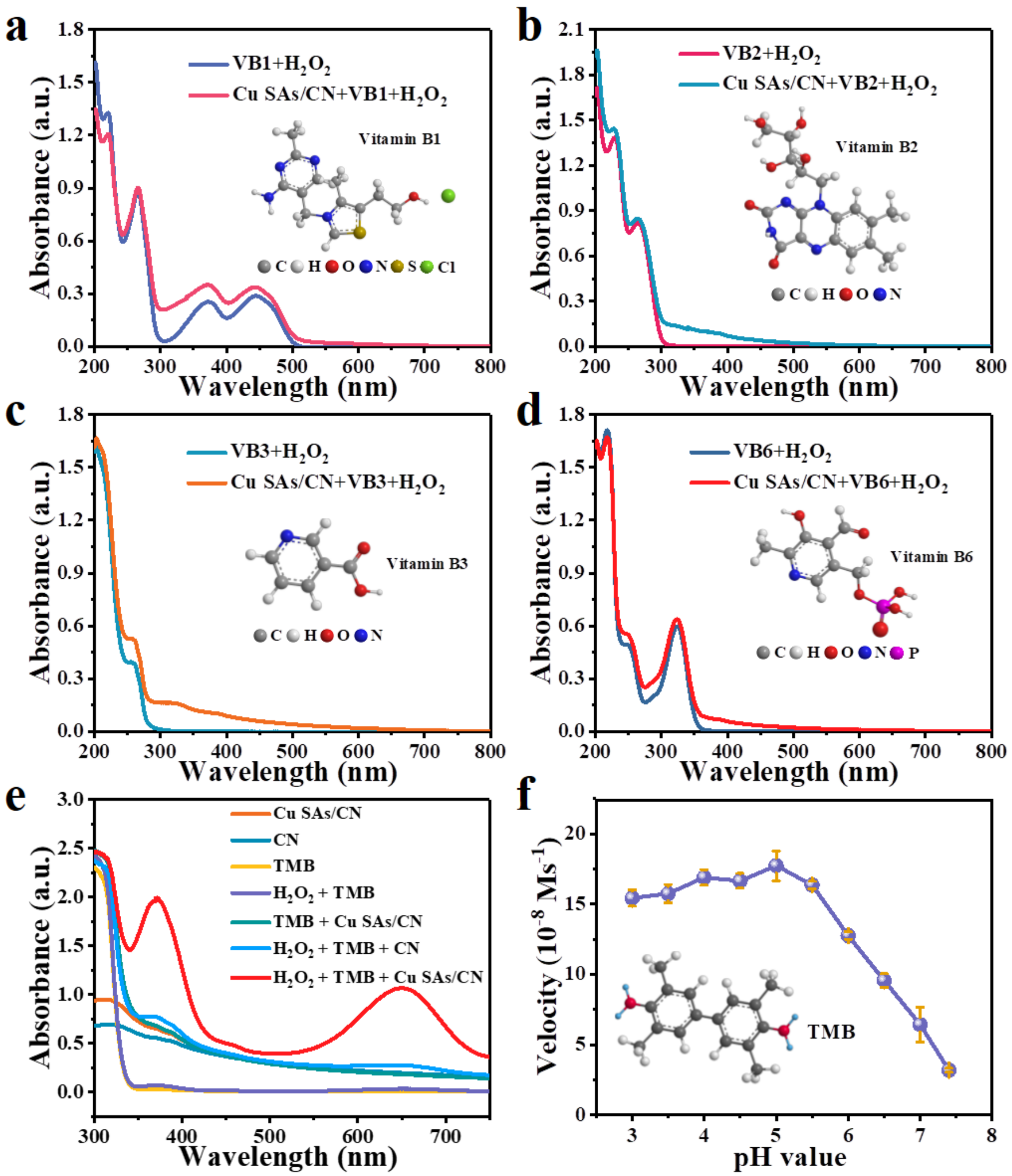

The selectivity for the APX-like of Cu SAs/CN towards AsA was examined by employing few relevant Vitamin complexes (VB1, VB2, VB3 and VB6) and the

typical peroxidase reducing substrate TMB to replace AsA. As depicted in the above panel (a) to (d), after incubating few relevant Vitamin complexes (VB1, VB2, VB3 and VB6) with Cu SAs/CN and H_2O_2 in PBS solution (10 mM, pH = 7.4), the corresponding characteristic absorbance peaks of the vitamin complexes show a negligible change. Whereas after introducing the TMB in acetate buffer solution (0.2 M, pH = 4.5), a distinct characteristic absorbance of oxidized TMB (blue) at 652 nm in keeping with that of HRP[21] can be observed in panel (e), which demonstrates the intrinsic peroxidase-like performance of Cu SAs/CN. However, the peroxidase mimic performance of Cu SAs/CN was gradually decreased when increasing the pH value. With a view to the subsequent biological applications, when performed the peroxidase-like reaction in the PBS solution (10 mM, pH = 7.4), negligible peroxidase catalytic activity can be founded based on Cu SAs/CN. Thus these results confirmed the high selectivity of APX-like performance of Cu SAs/CN towards AsA under the neutral environment.

Figure S21. Schematic illustration of the process of AsA oxidized by Cu SAs/CN with intrinsic APX-like activity in the presence of H_2O_2. And the mechanism of fluorescence-based sensing AsA in which OPDA is applied as the probe.

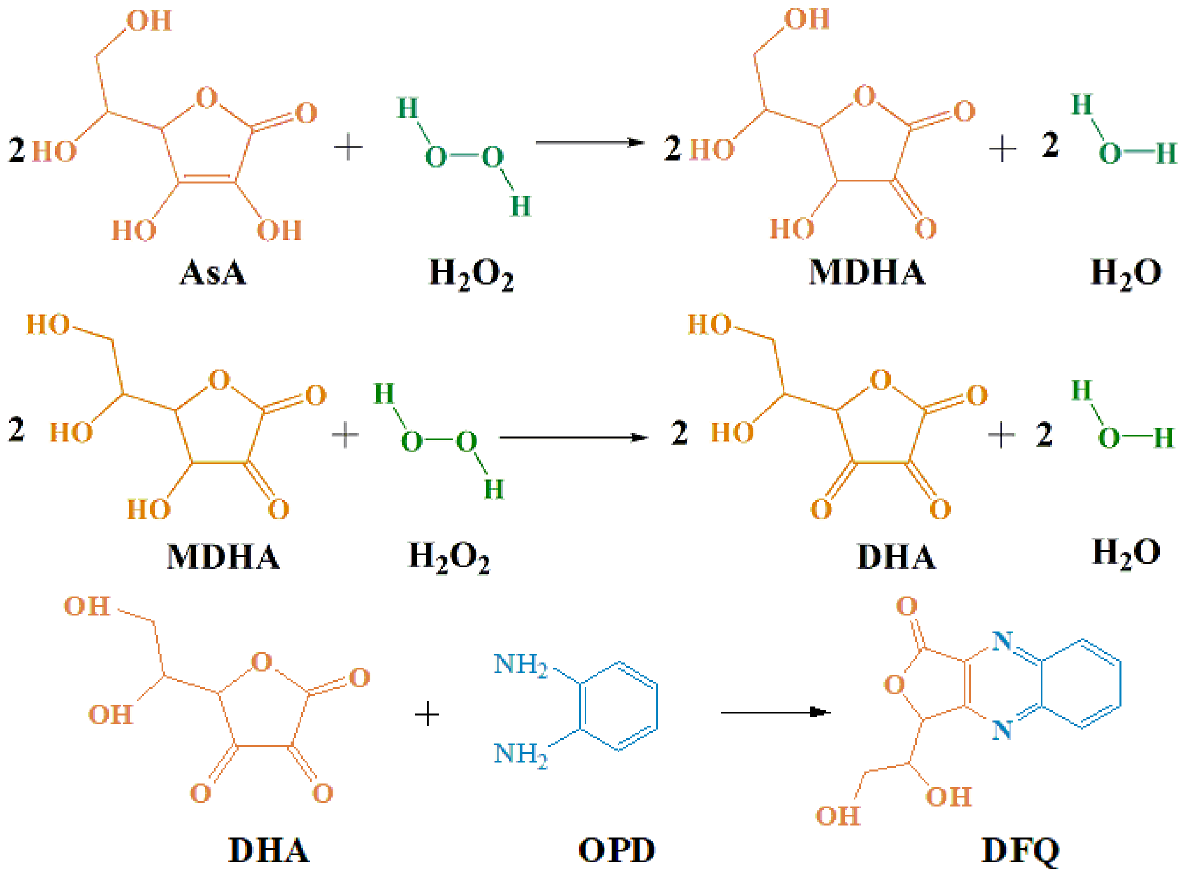

Figure S22. The corresponding calibration plots were constructed using the fluorescence intensity at 430 nm.

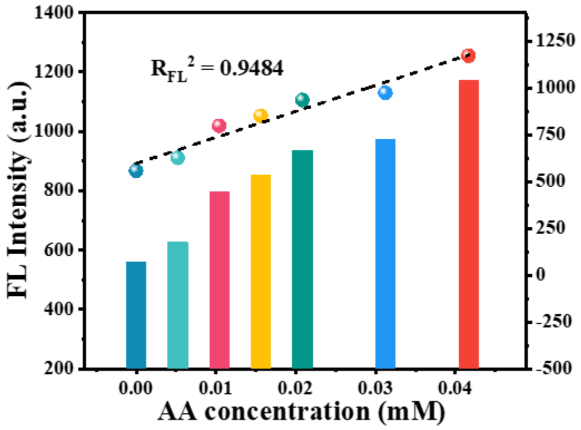

Figure S23. The reaction process of Cu SAs/CN for activating H_2O_2 through heterolytic (red line) and hemolytic path (blue line).

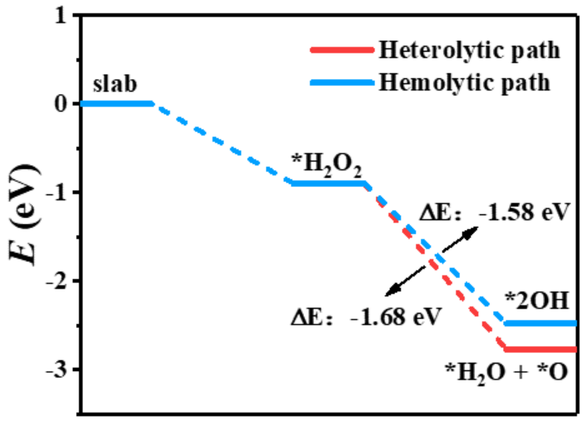

The dissociation process of H_2O_2 molecules on Cu SAs/CN has also been investigated by DFT. As shown in the above spectra, two possible dissociation path has been listed. The absorbed H_2O_2 molecules dissociate and forming 2*OH by hemolytic. Whereas, H_2O_2 dissociates *via* a heterolytic route, resulting in the generation of *H_2O + *O. The energy barrier for the hemolytic (E_a:-1.58 eV) is higher than that for the heterolytic path (E_a:-1.68 eV). Therefore, H_2O_2 molecules are thermodynamically decomposed by Cu SAs/CN through the heterolytic path.

Figure S24. (a) The dynamic light scattering (DLS) spectra of Cu SAs/CN. (b) The serum stability of Cu SAs/CN after incubation in DMEM medium supplemented with 10% FBS with varied time.

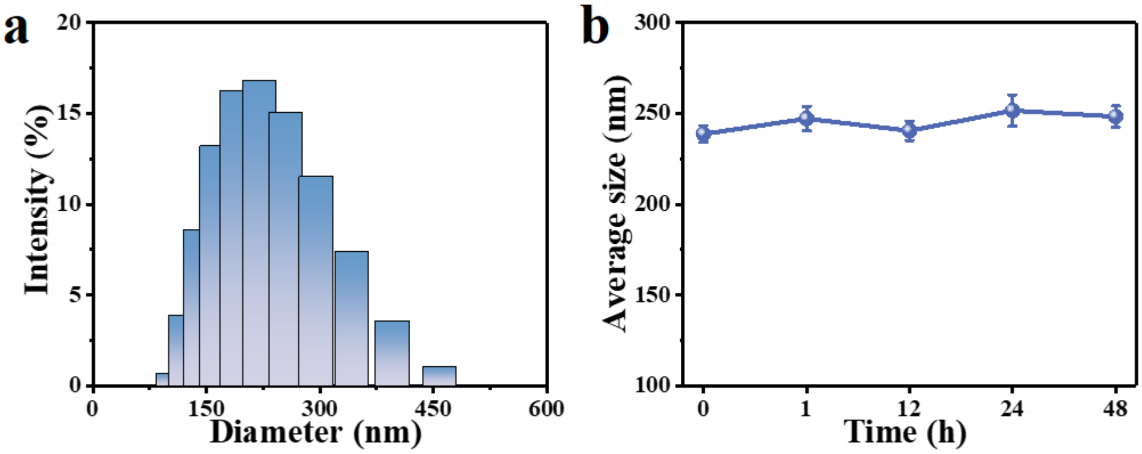

DLS measurement and Zeta potential were performed to investigate the diameters distribution and the surface charge of Cu SAs/CN. As depicted in the DLS spectra in panel (a), the diameters of Cu SAs/CN were mainly ranged from 150 to 300 nm. Meanwhile, the Zeta potential of Cu SAs/CN was determined to be -15.25 mV. After 48 h incubation, the DLS diameters of Cu SAs/CN exhibited negligible fluctuations in panel (b). Taken together, the negative Zeta potential and serum stability result of Cu SAs/CN suggested their superior stability.

Figure S25. Cellular uptake capacity of Cu SAs/CN. (a), (c) Fluorescence images and (b), (d) merged images of HeLa cells incubated without and with Cu SAs/CN for 24 h, respectively. CLSM was then employed to manifest the cell uptake capability with an excitation wavelength of 405 nm and an emission filter of 410-510 nm. The scale bar: 50 μm. (e) Fluorescence image of Cu SAs/CN.

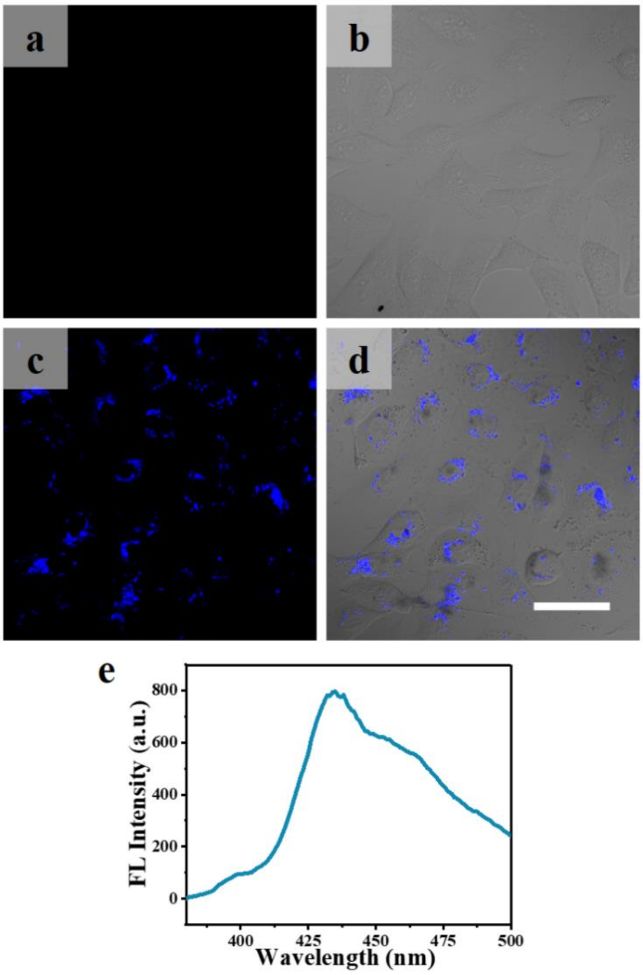

As shown in panel (e), Cu SAs/CN emits fluorescence located around 435 nm with an excitation wavelength of 405 nm. This specific fluorescence is in favor of visually observing the uptake of cells. Based on this characteristic, the cell uptake capacity assay of Cu SAs/CN has been conducted. For the cells without being treated with Cu SAs/CN, no significant fluorescence was detected in (a), (b). Comparatively, the blue

fluorescence refers to the Cu SAs/CN obviously emerged inside of the HeLa cells instead of enriching in the surrounding cell membrane, suggesting that Cu SAs/CN was phagocytosed by HeLa cells. Therefore, the above results further convinced that Cu SAs/CN can be internalized by HeLa cells. And Cu SAs/CN is applied to protect the cells by its function inside of cells.

References

1. H. B. Li, M. H. Yu, F. X. Wang, P. Liu, Y. Liang, J. Xiao, C. X. Wang, Y. X. Tong, *Nat. Commun.* **2013,** *4*, 1894.

2. H. B. Li, Y. Q. Gao, C. X. Wang, G. W. Yang, *Adv. Energy Mater.* **2015,** *5*, 1401767.

3. L. Z. Gao, J. Zhuang, L. Nie, J. B. Zhang, Y. Zhang, N. Gu, T. H. Wang, J. Feng, D. L. Yang, S. Perrett, X. Y. Yan, *Nat. Nanotechnol.* **2007,** *2*, 577-583.

4. B. Jiang, D. M. Duan, L. Z. Gao, M. J. Zhou, K. L. Fan, Y. Tang, J. Q. Xi, Y. H. Bi, Z. Tong, G. F. Gao, N. Xie, A. F. Tang, G. H. Nie, M. M. Liang, X. Y. Yan, *Nat. Protoc*. **2018,** *13*, 1506-1520.

5. C. Y. Wu,; X. G. Han,; W. Feng,; Z. L. Liu,; L. S. Chen,; B. G. Zhou,; Y. Chen,; J. L. Shi, *Chem. Eng. J.* **2021,** *411*, 128543.

6. W. G. Liu, L. L. Zhang, X. Liu, X. Y. Liu, X. F. Yang, S. Miao, W. T. Wang, A. Q. Wang, T. Zhang, *J. Am. Chem. Soc.* **2017,** *139*, 10790-10798.

7. Y. Xu, J. Xue, Q. Zhou, Y. J. Zheng, X. H. Chen, S. Q. Liu, Y. F. Shen, Y. J. Zhang, *Angew. Chemie., Int. Ed*. **2020,** *59*, 14498-14503.

8. W. A. Boggust, H. McCauley, *Br. J. Cancer* **1978,** *38,* 100-05.

9. Z. Q. Lu, T. Takano, S. K. Liu, *Biotechnol. Lett.* **2005,** *27*, 63-67.

10. T. Takeda, K. Yoshimura, T. Ishikawa, S. Shigeoka, *Biochimie* **1990,** *80,* 295-301.

11. K. S. Yadav, P. Yadav, S. Sharma, S. K. Khare, *Int. J. Biol. Macromol.* **2019,** *122*, 962-968.

12. S. Kitajima, M. Ueda, S. Sano, C. Miyake, T, Kohchi, K.-I. Tomizawa, S. Shigeoka, A. Yokota,

Biosci. Biotechnol. Biochem. **2002,** *66*, 2367-2375.

13. T. P. Barrows, T. L. Poulos, *Biochem.* **2005,** *44*, 14062-14068.

14. C. L. MetCalfe, M. Ott, N. Patel, K. Singh, S. C. Mistry, H. M. Goff, E. L. Raven, *J. Am. Chem. Soc.* **2004,** *126*, 16242-16248.

15. M. O. Fouad, A. Essahibi, L. Benhiba, A. Qaddoury, *Span. J. Agric. Res.* **2014,** *12*, 763-771.

16. V. R. Patel, N. Bhatt, *Arabian J. Chem.* **2020,** *13,* 8783-8792.

17. J. X. Ni, D. M. Liu, W. Wang, A. W. Wang, J. L. Jia, J. Y. Tian, Z. P. Xing, *Chem. Eng. J.* **2021,** *419*, 129969.

18. B. Yan, C. Du, G. W. Yang, *Small* **2020,** *16*, 1905700.

19. Y. Chen, X. J. Wu, T. M. Chen, G. W. Yang, *ACS Appl. Mater. Interfaces* **2020,** *12,* 19357-19368.

20. C. Hu, F. Chen, Y. G. Wang, N. Tian, T. Y. Ma, Y. H. Zhang, H. W. Huang, *Adv. Mater.* **2021,** *33*, 2101751.

21. H. Wang, P. H. Li, D. Q. Yu, Y. Zhang, Z. Z. Wang, C. Q. Liu, H. Qiu, Z. Liu, J. S. Ren, X. G. Qu, *Nano lett.* **2018,** *18*, 3344-3351.
